# Effect of Local Anesthetics on Experimental Postoperative Adhesion: A Systematic Review and Meta-Analysis with Trial Sequential Analysis

**DOI:** 10.3390/medicina61122215

**Published:** 2025-12-15

**Authors:** Joon-hee Lee, Donghyun Lee, Hyun Kang

**Affiliations:** 1 Department of Integrative Engineering, Chung-Ang University Graduate School, Seoul 06974, Republic of Korea; 2 Department of Anesthesiology and Pain Medicine, Chung-Ang University College of Medicine, Seoul 06974, Republic of Korea

**Keywords:** anesthetics, local, animal experimentation, tissue adhesions, meta-analysis as topic, systematic review, trial sequential analysis

## Abstract

*Background and Objectives*: We performed a systematic review and meta-analysis using trial sequential analysis (TSA) to investigate the potential preventive postoperative antiadhesive effects of local anesthetics (LA). *Materials and Methods*: A comprehensive search was conducted using Ovid-MEDLINE, Ovid-EMBASE, Web of Science, and Google Scholar to identify animal studies that explored the postoperative antiadhesive effect of LA applied in the surgical area. The primary outcome was the macroscopic adhesion score, including adhesion quality, quantity, and total adhesion score, whereas the secondary outcome was the microscopic adhesion score, including adhesion severity, inflammation, and fibrosis. Certainty of evidence was assessed using a GRADE-adapted framework for animal studies. *Results*: The comprehensive analysis involved 227 rats across 6 animal studies, with 158 rats subjected to LA and the remaining 69 administered a placebo or received no treatment. For macroscopic adhesion score, LA were associated with reductions in the total adhesion score (standardized mean difference (SMD) −1.528; 95% confidence interval (CI) −2.081 to −0.976; I^2^ = 30.0%) and adhesion quality (SMD: −0.996; 95% CI −1.906 to −0.085; I^2^ = 72.6%), while no significant difference was observed in adhesion quantity (SMD −0.544; 95% CI −1.452 to 0.365; I^2^ = 77.6%). For the microscopic adhesion score, LA appeared to reduce adhesion severity (SMD −1.304; 95% CI −1.862 to −0.746; I^2^ = 31.7%) and fibrosis (SMD: −2.373; 95% CI −3.400 to −1.346; I^2^ = 60.4%), whereas the effect on inflammation was inconsistent. Across all macroscopic outcomes, TSA demonstrated that the accrued sample size was far below the required information size, and the certainty of evidence remained low to very low. Most included studies had unclear or high risks of bias, which reduces confidence in the synthesized estimates. *Conclusions*: LA may have a potential association with reduced postoperative adhesion formation; however, the certainty of evidence was low to very low, and TSA indicated insufficient required information size to draw firm conclusions.

## 1. Introduction

Postoperative adhesion is a physiological reparative phenomenon that occurs as a part of the natural healing process following surgery [1]. However, despite their beneficial intent, postoperative adhesions can result in significant complications, including mechanical intestinal obstruction, female infertility, challenges in reoperation, chronic postsurgical pain and paraplegia [2,3]. These complications contribute to an increased need for future surgeries, higher readmission rates, longer hospital stays, and higher medical expenses after surgery [4,5,6].

Adhesion is involved in various biological processes, and inflammation is a key factor in this process [1]. Surgical trauma triggers the body’s natural inflammatory response, releasing inflammatory mediators that promote cell migration and fibrin formation and facilitate tissue healing. However, under pathological conditions, this can lead to the formation of undesirable fibrous bands or adhesions.

Local anesthetics have anti-inflammatory properties that can potentially prevent postoperative adhesion [7]. This is achieved by blocking nerve impulses and inhibiting the release of neurotransmitters such as substance P and calcitonin gene-related peptide (CGRP), thereby reducing the production of inflammatory mediators. Moreover, their pain-relieving action at the surgical site improves patient comfort and mobility, indirectly affecting the response of the immune system to inflammation. Local anesthetics also activate the fibrinolytic system, leading to a reduction in factor VIII, plasminogen, and alpha-2-antiplasmin concentrations and inhibition of platelet aggregation [8]. Further, local anesthetics may reduce adhesion formation by attenuating the systemic surgical stress response and limiting the downstream pathways—such as hypoperfusion, excessive fibrinogenesis, and delayed fibrinolysis—that promote adhesions.

Despite many studies investigating the anti-adhesive effects of local anesthetics, the results have been inconsistent. Importantly, all currently available evidence derives exclusively from preclinical animal studies, and no clinical trials have evaluated these effects in humans. Substantial methodological heterogeneity—including differences in animal species, surgical models, adhesion scoring systems, and type and dose of local anesthetics—limits the generalizability of individual study results. Moreover, extrapolating these preclinical findings to human surgical practice is inherently challenging, as animal healing physiology, inflammatory responses, and adhesion biology differ meaningfully from those of humans. Therefore, any interpretation of clinical relevance must remain cautious.

To address these uncertainties, we conducted a systematic review and meta-analysis incorporating trial sequential analysis (TSA) to critically evaluate the available preclinical evidence. The primary outcome was the macroscopic adhesion score, which assesses adhesion formation based on quality (tenacity), quantity (extent), or total adhesion score. Microscopic parameters—including severity, inflammation, and fibrosis—were evaluated as secondary outcomes to capture tissue-level responses. This systematic review was designed to test the hypothesis that local anesthetic administration at the surgical site reduces macroscopic adhesion formation and improves microscopic parameters such as severity, inflammation, and fibrosis in experimental models. By evaluating these outcomes, our study aims to clarify whether local anesthetics demonstrate a potential anti-adhesive effect in preclinical settings.

## 2. Materials and Methods

### 2.1. Protocol and Registration

The methodological framework for this rigorous investigation—comprising a systematic review and meta-analysis with trial sequential analysis—was developed in line with PRISMA-P recommendations. The study protocol was registered in the PROSPERO database on 25 July 2023 (registration number: CRD42023445704; www.crd.york.ac.uk/Prospero, accessed on 25 August 2023). This systematic review and meta-analysis, including a trial sequential analysis evaluating the effects of local anesthetics on experimental postoperative adhesion, was conducted following the protocol outlined by the Cochrane Collaboration [9] and reported in accordance with PRISMA guidelines [10]. The methodological approach, including the implementation of trial sequential analysis, was informed by our previous work [11,12].

### 2.2. Eligibility Criteria

A systematic search was performed following the application of predefined inclusion and exclusion criteria. We included full-text reports of animal studies that compared the application of local anesthetics at the surgical site with a control group for the prevention of postoperative adhesion.

The PICO-SD framework for this review was defined as follows:

Animals/Population (P): Any animal species undergoing surgery

Intervention (I): Local anesthetics administered directly to the surgical site

Comparison (C): No exposure to active drugs (e.g., no local anesthetic administration; normal saline or distilled water)

Outcome (O): The primary outcome was the macroscopic adhesion score, including quality (tenacity), quantity (extent), and total adhesion score. The secondary outcome was the microscopic adhesion score, assessed by the severity of adhesion, inflammation, and fibrosis.

Study Design (SD): Controlled studies with distinct treatment groups

The following were excluded: (1) studies without animal subjects (e.g., ex vivo, in vitro, or human studies); (2) studies evaluating interventions other than local anesthetics; (3) studies lacking relevant outcome data; (4) studies without a control group; and (5) review articles, case reports, case series, letters, commentaries, and conference proceedings.

Because the objective of this systematic review was to synthesize the anti-adhesive effects of local anesthetics across preclinical settings, we included various surgical models. Although these models differ in baseline adhesion risk, their mechanisms of action (anti-inflammatory and fibrinolytic effects) are not model-dependent. Therefore, the inclusion of multiple models was considered appropriate for evaluating the generalizable effects of local anesthetics across experimental conditions.

### 2.3. Literature Search

In July 2023, two independent researchers conducted systematic literature searches across multiple databases, including Ovid-MEDLINE, Ovid-EMBASE, Web of Science, and Google Scholar. Because an additional eligible study was published after the initial search [13], the search was updated in September 2025 to ensure complete and up-to-date coverage of relevant evidence, representing a minor deviation from the original protocol. The detailed search strategy, incorporating free-text terms, Medical Subject Headings (MeSH), and EMTREE terminology, is provided in the Supplementary Information File.

All retrieved records were imported into EndNote (version 21; Clarivate, Philadelphia, PA, USA), and duplicate entries were removed. To further ensure comprehensive identification of relevant studies, we manually screened the reference lists of all included articles until no additional eligible studies were found. No language or publication-date restrictions were applied. Although non-English articles were eligible for inclusion, none of the full-text studies meeting the criteria were written in languages other than English. Had eligible non-English studies been identified, translation would have been conducted with support from domain experts at Chung-Ang University.

### 2.4. Study Selection

Two investigators independently screened the titles and abstracts of all retrieved records. Any study judged as potentially eligible by either reviewer was advanced to full-text evaluation. Conference proceedings were also examined. To avoid duplication, studies originating from the same authors, institutions, or countries were cross-checked. Full-text articles that met the inclusion criteria were then independently assessed by both investigators, with any discrepancies resolved through discussion or, when necessary, consultation with a third investigator.

### 2.5. Data Extraction

Data extraction for this systematic review and meta-analysis with trial sequential analysis followed the approach used in our previous work [11,12]. Two investigators independently extracted all relevant information using standardized extraction forms, and any discrepancies were resolved through consensus. When disagreements occurred—particularly regarding values derived with Plot Digitizer—both reviewers re-examined the original graphical data until agreement was reached. A subsequent cross-validation step was then performed.

Local anesthetics administered at the surgical site were classified as the LA group, irrespective of the specific agent, dose, or delivery method. Placebo controls (saline or distilled water) were pooled with no-treatment controls to form the control group, as none possess pharmacologic activity and all have been consistently treated as biologically inactive comparators in prior preclinical meta-analyses [11,12]. When a study included multiple LA groups that were eligible for comparison, these were combined for the analysis of the overall effect.

The standardized extraction form collected the following information: (1) article title, (2) first author, (3) journal name, (4) publication year, (5) animal species, (6) type of surgery, (7) control-group intervention (no treatment, saline, or distilled water), (8) kinds and doses of local anesthetics, (9) definition of macroscopic adhesion score (ex. quality (or tenacity of adhesion), quantity (or extent of adhesion), or total adhesion score), (10) definition of microscopic adhesion score (ex. severity, inflammation, and fibrosis), (11) degree of macroscopic adhesion, (12) the degree of microscopic adhesion, and (13) informations to assess methodological quality of studies.

Data were primarily extracted from tables or textual descriptions. If such information was unavailable, the study authors were contacted. When data could not be obtained directly, missing values were extracted from graphical sources using the open-source software Plot Digitizer (version 2.6.8; http://plotdigitizer.sourceforge.net, accessed on 1 September 2023).

Among the included studies, only two studies required numerical extraction from graphical presentations [14,15]. In the study of Suckow et al., the graphs represented whole-animal counts, enabling exact integer extraction without measurement uncertainty [14]. The studies by Parsa, Ozturk, and Kesici et al. reported all outcomes numerically in tables or text [13,16,17], and we had direct access to the raw dataset for the Choi et al. study [18]. Thus, only the study of Yuzbasioglu et al. contained data in which digitization could introduce potential error [15].

### 2.6. Methodological Quality and Publication

The methodological quality assessment for this systematic review and meta-analysis with trial sequential analysis was performed following the approach outlined in our previous studies [11,12]. Five domains were evaluated: (1) random allocation to treatment and control groups, (2) husbandry conditions (including light–dark cycle, temperature, water access, and environmental enrichment), (3) adherence to animal welfare regulations, (4) potential conflicts of interest, and (5) publication in a peer-reviewed journal. Two investigators independently assessed each study, and any disagreements were settled through adjudication by a third reviewer (Kang H).

In addition to these domains, we assessed methodological rigor using SYRCLE’s Risk of Bias (RoB) tool, which is specifically tailored for evaluating bias in animal intervention studies [19].

### 2.7. Statistical Analyses

Statistical analyses for this systematic review and meta-analysis with trial sequential analysis were carried out in accordance with the methods used in our previous studies [11,12]. Ad hoc summary tables were constructed to present key characteristics of the included studies and to address essential questions aligned with the review objectives. After data extraction, the feasibility of conducting a meta-analysis was evaluated. Two investigators independently entered all extracted data into the analysis software. For each outcome, standardized mean differences (SMDs) and 95% confidence intervals (CIs) were calculated. SMDs were computed as the mean value of the LA group minus that of the control group, such that negative SMDs indicate reduced adhesion formation in the LA group. Between-study heterogeneity was assessed using Cochran’s Q, Higgins’s I^2^ statistics, and τ (Tau) [20]. A *p*-value < 0.10 for the chi-square test or an I^2^ > 50% was considered indicative of heterogeneity. When heterogeneity was present—or when fewer than ten studies were available—the t-statistic–based Hartung–Knapp–Sidik–Jonkman method was applied in place of the Z-test to reduce error rates [21].

A subgroup analysis was performed according to the type of local anesthetics. Additional subgroup analyses (e.g., by surgical model or species) were not conducted because only six studies were available, resulting in cell sizes too small for meaningful interpretation. Sensitivity analyses were undertaken by sequentially removing each study to assess its influence on the overall pooled effect. Meta-regression was also performed using dose per coefficient, calculated as the administered dose divided by the partition coefficient (n-octanol/water) [22]. The partition coefficient reflects the lipid-solubility–dependent potency of each local anesthetic, allowing standardization of physicochemical potency differences across studies [22,23].

When studies reported outcomes as median (P25–P75), median (range), or mean with standard error, mean values and standard deviations were derived using established transformation methods [24].

Because fewer than ten studies were available for each outcome, formal assessment of publication bias (e.g., funnel plot asymmetry, Egger’s test) was not performed, as such analyses are statistically unreliable with small study numbers. However, the potential for publication bias cannot be completely excluded.

In the forest plots, each individual study is displayed as a filled square proportional to its sample size, with horizontal lines representing the 95% CI. The pooled estimate and its uncertainty are illustrated by a diamond. The numerical weights assigned to each study are provided in Supplementary Table S1 for full transparency.

### 2.8. Trial Sequential Analysis

Additionally, we conducted a TSA of the macroscopic adhesion score—including quantity, quality, and total adhesion score—to calculate the required information size (RIS) and determine whether the accumulated evidence was conclusive [25]. A random-effects model was used to generate the cumulative Z-curve, and TSA was performed while maintaining an overall 5% risk of type I error. In the TSA graph, the upper and lower red curves denote the trial sequential monitoring boundaries for benefit and harm, respectively. The horizontal dotted line represents conventional statistical significance, and the triangular red boundaries on the right indicate futility. The *x*-axis represents the required information size.

Crossing a trial sequential monitoring boundary or entering the futility area with the cumulative Z-curve indicates that the available evidence is sufficient to confirm or refute the anticipated intervention effect, suggesting that additional trials may no longer be necessary. Conversely, if the Z-curve does not cross any monitoring boundary and the RIS is not achieved, the evidence remains inconclusive, and further studies are required.

For the macroscopic adhesion score, we used the observed standard deviation (SD), a mean difference corresponding to SD/3, an alpha level of 5% for all outcomes, a beta of 20%, and the observed diversity derived from the included trials. These parameters were entered into the TSA software to compute the monitoring boundaries, and RIS was required to obtain adequately powered conclusions.

In this systematic preview, the cumulative Z-curve for quality and total adhesion score crossed the conventional significance boundary but did not cross the trial sequential monitoring boundary, while the accrued sample size was far below the RIS. This pattern suggests that the nominal statistical significance identified in the conventional random-effects models should be interpreted cautiously, as the evidence remains inconclusive without meeting the trial sequential monitoring boundary or RIS in TSA. The TSA therefore suggests that current data are insufficiently powered and that additional well-designed studies are required before any definitive conclusion can be drawn.

Traditional meta-analyses were performed using Comprehensive Meta-Analysis software (version 2.0; Biostat, Englewood, NJ, USA), and trial sequential analyses were conducted using TSA software (version 0.9.5.10; Copenhagen Trial Unit, Centre for Clinical Intervention Research, Copenhagen, Denmark).

### 2.9. Certainty of Evidence

The certainty of evidence for each outcome was evaluated using the framework proposed by Hooijmans et al. [26], which adapts the GRADE approach for use in preclinical animal research. Certainty was qualitatively categorized as high, moderate, low, or very low, based on considerations of risk of bias, inconsistency, indirectness, imprecision, and potential publication bias.

## 3. Results

### 3.1. Study Selection

The search of Ovid-MEDLINE, Ovid-EMBASE, Web of Science, and Google Scholar yielded 563 studies, supplemented by eight additional studies identified through manual searching. After adjusting for duplicates (n = 6), 565 studies were included. Of these, 548 were excluded after reviewing their titles and abstracts because they were not relevant. At this stage of study selection, the kappa value for selecting studies between the two reviewers was 0.807. The full texts of the remaining 17 studies were reviewed in detail, and 11 studies were excluded for the following reasons: human data (n = 4) [27,28,29,30], were not compared with local anesthetics (n = 3) [31,32,33], did not report appropriate outcomes (n = 3) [34,35,36], was a review article (n = 1) [37]. The kappa value for the articles selected by the two investigators was 1.000.

**Figure 1:**
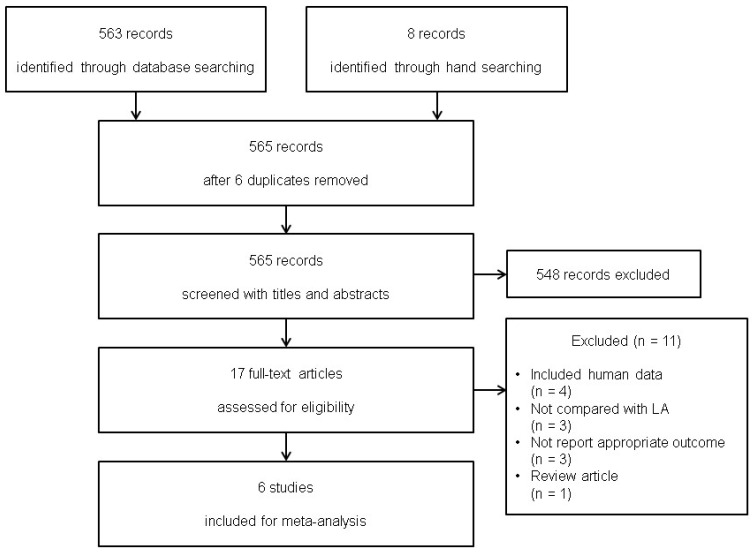
Flow diagram showing the number of abstracts and articles identified and evaluated during the review.

Thus, six studies (including 227 animals) with 69 animals assigned to the control group and 158 animals receiving local anesthetics met the inclusion criteria and were included in this systematic review and meta-analysis with trial sequential analysis (Figure 1).

The characteristics of the included studies are summarized in Table 1.

The types of surgeries performed included laparotomy [14,15,16,17,19] and the incisional pain model [18]. Wistar rats [15], Wistar-Albino rats [17], and Sprague Dawley rats [14,16,18,19] were used. For the control group, no irrigation [14,15] or both irrigation and normal saline [13,16,17,18] was applied. For the experimental group, lidocaine [15,16,17,18] lidocaine and prilocaine (EMLA) [15], bupivacaine [13,14,16,17], levobupivacaine [13] were used in the experimental group.

### 3.2. Macroscopic Adhesion Score

Because the included studies used heterogeneous scoring systems, we organized macroscopic outcomes into three prespecified components—quality (tenacity), quantity (extent), and total adhesion score—regardless of the original terminology. Four studies measured macroscopic adhesion score [13,14,15,16,17]. Of these, two studies [16,17] reported outcome using quality (detached with mild, moderate, and severe traction), quantity (percentage of adhesions in the total abraded area), and total adhesion score (sum of scores); one study [14] reported outcomes using the extent and tenacity of adhesion; and one study [15] reported outcome using scores according to the method of Bothin et al. [38] and one study [13] reported outcome using the mehod of Roohbakhsh et al. [39] (Supplementary Table S2).

**Figure 2:**
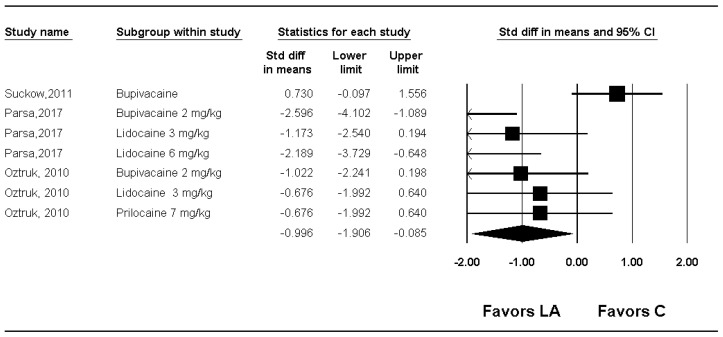
Forest plot showing the effect of local anesthetics compared with control on the quality of adhesion. Each trial is shown as a filled square proportional to sample size, with the 95% confidence interval (CI) depicted as a horizontal line; the diamond indicates the pooled effect and its uncertainty. C, control; LA, local anesthetic [14,16,17].

The quality (or tenacity) of adhesion was compared in three studies [14,16,17]. Quality was significantly lower in the group LA compared with group C (SMD −0.996; 95% CI −1.906 to −0.085; P_chi_^2^ = 0.001; I^2^ = 72.6%; τ = 1.036) (Figure 2, Table 2, Supplementary Table S1).

However, sensitivity analysis showed that the statistical significance was largely driven by the study by Suckow et al. [14]; removal of this single study resulted in loss of significance (Supplementary Figure S1). This indicates that the pooled effect for quality is not robust to the influence of individual studies.

When subgroup analysis was performed for kinds of local anesthetics used, quality was significantly lower in the group LA than in the group C for lidocaine (SMD −1.190; 95% CI −1.819 to −0.560; P_chi_^2^ = 0.147; I^2^ = 47.8%; τ = 0.549) (Supplementary Figure S2, Supplementary Table S1), but there was no evidence of differences for bupivacaine (SMD −0.928; 95% CI −2.814 to 0.959; P_chi_^2^ < 0.001; I^2^ = 91.1%; τ = 1.587) and prilocaine (SMD −0.676; 95% CI −1.577 to 0.226) (Supplementary Figures S3 and S4, Supplementary Table S1). In the meta-regression analysis, no significant relationship was observed between the dose per coefficient and quality (slope −0.18336; 95% CI −0.88851 to 0.52180; *p* = 0.61031) (Supplementary Figure S5).

**Figure 3:**
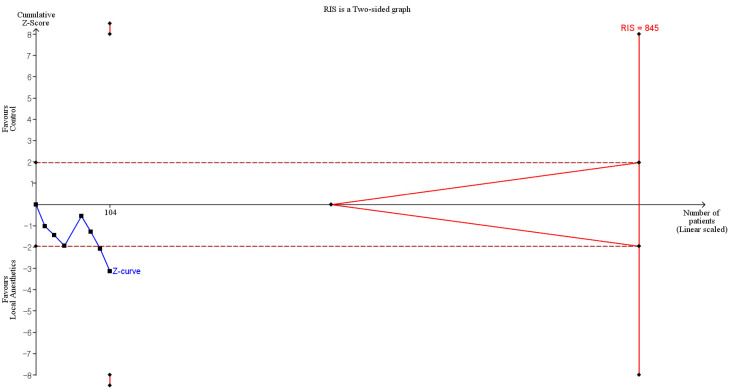
Trial sequential analysis (TSA) showing the effect of local anesthetics compared with control on the quality of adhesion. The uppermost and lowermost solid red curves denote the trial sequential monitoring boundaries for benefit and harm, respectively. The horizontal dotted red line marks the conventional significance threshold. Triangular red lines at the right indicate the futility boundaries. The vertical solid red line indicates the required information size. The solid blue line is the cumulative z-curve. Numbers on the *x*-axis indicate the required information size.

TSA indicated that only 12.3% (104 of 845 animals) of the RIS was accrued, and although the cumulative Z-curve crossed the conventional boundary, it did not cross the trial sequential monitoring boundary, suggesting insufficient evidence to confirm a firm treatment effect (Figure 3, Table 2).

The quantity (or extent) of adhesion was compared in three studies [31,32,33]. There was no evidence of difference for quantity between group LA and group C (SMD −0.544; 95% CI −1.452 to 0.365; P_chi_^2^ = 0.001; I^2^ = 77.6%; τ = 1.044) (Supplementary Figure S6, Table 2, Supplementary Table S1). Sensitivity analysis again showed that removing the Suckow et al. study [14] altered the significance pattern, indicating that this outcome was also highly sensitive to a single study. (Supplementary Figure S7). When subgroup analysis was performed for kinds of local anesthetics used, there was no evidence of differences for bupivacaine (SMD: −0.418; 95% CI −2.261 to 1.426; P_chi_^2^ < 0.001; I^2^ = 87.5%; τ = 1.519), lidocaine (SMD: −0.895; 95% CI −1.980 to 0.189; P_chi_^2^ = 0.050; I^2^ = 66.7%; τ = 0.782) and prilocaine (SMD: 0.000; 95% CI −0.877 to 0.877) (Supplementary Figures S8–S10, Supplementary Table S1) In the meta-regression analysis, no significant relationship was observed between dose per coefficient and quantity (Slope −0.11901; 95% CI −0.79985 to 0.56184; *p* = 0.73191) (Supplementary Figure S11).

TSA showed that only 13.5% (104 of 770 animals) of the RIS was reached, and the cumulative Z-curve crossed neither the conventional nor the trial sequential monitoring boundary, indicating insufficient accumulated information (Supplementary Figure S12).

**Figure 4:**
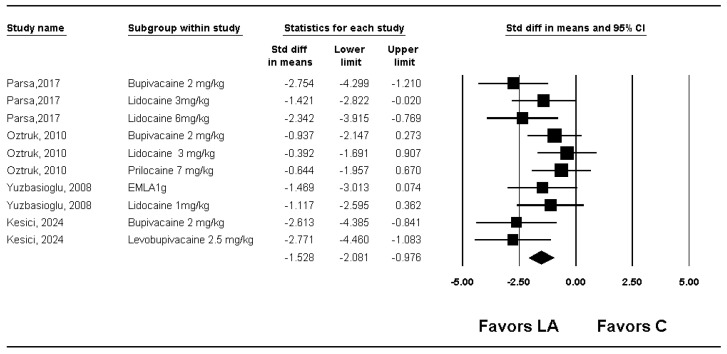
Forest plot showing the effect of local anesthetics on compared with control on the total adhesion score. Each trial is shown as a filled square proportional to sample size, with the 95% confidence interval (CI) depicted as a horizontal line; the diamond indicates the pooled effect and its uncertainty. C, control; LA, local anesthetic [13,15,16,17].

The total adhesion scores were compared in four studies [13,15,16,17]. The total adhesion score was significantly lower in the group LA compared with group C (SMD −1.528; 95% CI −2.081 to −0.976; P_chi_^2^ = 0.169; I^2^ = 30.0%; τ = 0.487) (Figure 4, Table 2, Supplementary Table S1).

By performing a sensitivity analysis by removing one study at a time, no changes were observed in the significance of the results (Supplementary Figure S13). When subgroup analysis was performed for kinds of local anesthetics used, total adhesion score was significantly lower in the group LA than in the group C for lidocaine (SMD −1.252; 95% CI −2.107 to −0.397; P_chi_^2^ = 0.072; I^2^ = 57.0%; τ = 0.430), bupivacaine (SMD: −2.010; 95% CI −3.480 to −0.541; P_chi_^2^ = 0.018; I^2^ = 75.0%; τ = 1.114), levobupivacaine (SMD: −2.613; 95% −4.385 CI to −0.841) and EMLA (SMD: −1.619; 95% CI −2.923 to −0.315) (Supplementary Figures S14–S17, Supplementary Table S1), but there was no evidence of differences for prilocaine (SMD: −0.597; 95% CI −1.493 to 0.299) (Supplementary Figure S18). In the meta-regression analysis, no significant relationship was observed between the dose per coefficient and total adhesion score (slope −0.31782; 95% CI −0.97651 to 0.34086; *p* = 0.34430) (Supplementary Figure S19).

**Figure 5:**
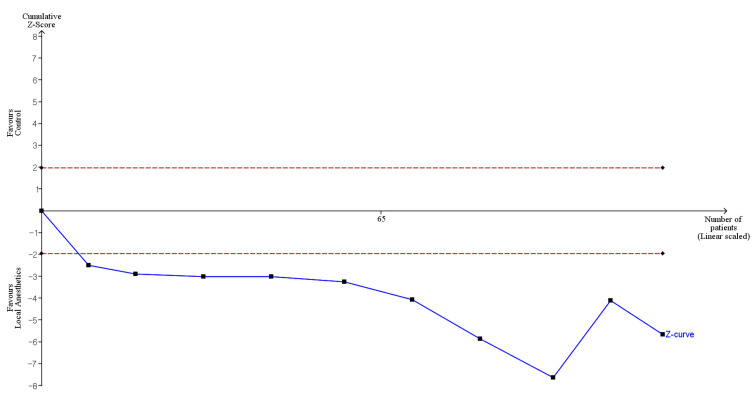
Trial sequential analysis (TSA) showing the effect of local anesthetics compared with control on the total adhesion score. The horizontal dotted red line marks the conventional significance threshold. The solid blue line is the cumulative z-curve. Numbers on the *x*-axis indicate the required information size.

TSA showed that only 4.1% (119 of 2936 animals) of the RIS was reached. Although the cumulative Z-curve crossed the conventional boundary, it did not cross the trial sequential monitoring boundary, confirming that the evidence remains inconclusive despite nominal statistical significance (Figure 5, Table 2).

### 3.3. Microscopic Adhesion Score

Two studies reported severity, defined by neovascularization and vessel-wall characteristics [16,17], and one study reported outcomes using inflammation and fibrosis [18]. One study reported inflammation descriptively [14]. Because only one to two studies contributed data to each microscopic outcome, these results should be interpreted as preliminary.

**Figure 6:**
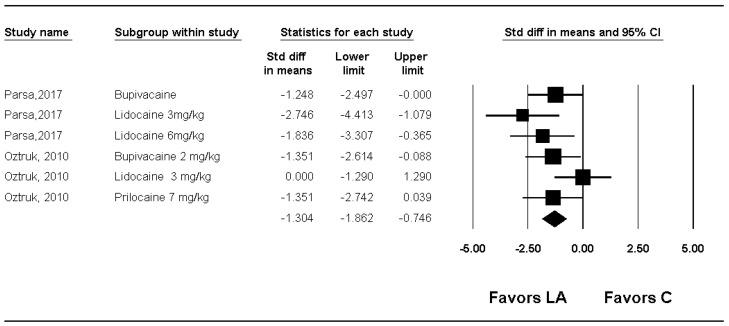
Forest plot showing the effect of local anesthetics compared with control on the severity of adhesion. Each trial is shown as a filled square proportional to sample size, with the 95% confidence interval (CI) depicted as a horizontal line; the diamond indicates the pooled effect and its uncertainty. C, control; LA, local anesthetic [16,17].

Severity significantly lower in the group LA compared with group C (SMD −1.304; 95% CI −1.862 to −0.746; P_chi_^2^ = 0.198; I^2^ = 31.7%; τ = 0.477) (Figure 6, Table 2, Supplementary Table S1)

By performing a sensitivity analysis by removing one study at a time, there were no changes in the significance of the results. (Supplementary Figure S20) When subgroup analysis was performed for kinds of local anesthetics used, severity was significantly lower in the group LA than in the group C for bupivacaine (SMD: −1.404; 95% CI −2.096 to −0.712; P_chi_^2^ = 0.881; I^2^ = 0.0%; τ = 0.0) (Supplementary Figure S21, Supplementary Table S1) and prilocaine (SMD: −1.351; 95% CI −2.323 to −0.380) (Supplementary Figure S22), but there was no evidence of differences for lidocaine (SMD −1.534; 95% CI −3.314 to 0.246; P_chi_^2^ = 0.001; I^2^ = 84.9%; τ = 1.443) (Supplementary Figure S23, Supplementary Table S1) In the meta-regression analysis, no significant relationship was observed between dose per coefficient and severity (Slope 0.21250; 95% CI −0.51396 to 0.93896; *p* = 0.56643) (Supplementary Figure S24).

For inflammation, there was no evidence of differences between group C and group LA (SMD: −1.833; 95% CI −4.017 to 0.350; P_chi_^2^ < 0.001; I^2^ = 90.9%; τ = 2.370) (Supplementary Figure S25, Table 2, Supplementary Table S1), with the substantial heterogeneity reflecting variation in histologic techniques, sampling depths, and inflammatory scoring systems. However, in the meta-regression analysis, the dose per coefficient was significantly correlated with inflammation (slope 3.66498; 95% CI 2.19376–5.13621; *p* < 0.00001) (Supplementary Figure S26).

Suckow et al. reported that a moderate number of acute inflammatory cells was observed in the bupivacaine-loaded group compared to mild-to-moderate chronic inflammation characterized by infiltrating macrophages in the bupivacaine-non-loaded group [14].

Fibrosis was significantly lower in the group LA compared with group C (SMD: −2.373; 95% CI −3.400 to −1.346; P_chi_^2^ = 0.039; I^2^ = 60.4%; τ = 0.909) (Supplementary Figure S27, Table 2, Supplementary Table S1). Nonetheless, this analysis relied on only two studies applying different fibrosis grading criteria, limiting comparability and increasing uncertainty. In the meta-regression analysis, the dose per coefficient was significantly correlated with fibrosis (slope, 2.27028; 95% CI, 0.79318–3.74738; *p* = 0.00259) (Supplementary Figure S28).

### 3.4. Side Effect

None of the included studies reported side effects associated with local anesthetic treatment; however, because adverse events were not systematically evaluated, the absence of reporting cannot be interpreted as evidence of safety.

### 3.5. Methodological Quality

A summary of the methodological quality assessment for each study is presented in Supplementary Table S3. The methodological quality scores ranged from 4 to 5. Two studies did not describe the method of random allocation [13,14], and one study did not report the husbandry conditions [17]. Each article was independently evaluated by two reviewers and scored on a 0-to-5 scale.

Supplementary Table S4 presents the risk-of-bias assessment performed using SYRCLE’s Risk of Bias (RoB) tool. Because the SYRCLE tool does not provide a validated method for calculating an overall risk-of-bias rating, we did not generate a composite score to avoid introducing subjective weighting. Nevertheless, several key domains—including allocation-sequence generation, allocation concealment, and blinding of investigators or outcome assessors—were frequently marked as “not described”. This predominance of unclear risk reflects substantial methodological uncertainty across the included studies and reduces confidence in the reliability and internal validity of the preclinical evidence.

### 3.6. Certainty of Evidence

The certainty of evidence, assessed using a preclinical GRADE-based framework, ranged from low to very low across outcomes (Supplementary Table S5).

## 4. Discussion

The current systematic review and meta-analysis with trial sequential analysis found that local anesthetics have beneficial effects in preventing postoperative adhesion quality, total adhesion score, severity, and fibrosis. However, these findings should be interpreted cautiously because several outcomes were substantially influenced by a single study [14], and sensitivity analyses showed that removing this study altered the statistical significance for both quality and quantity. This indicates that the robustness of the findings is limited and should be interpreted with caution. In addition, although some pooled estimates reached statistical significance, TSA consistently demonstrated that the pooled estimate did not reach statistical significance when considering inflation of type 1 error, and the accrued information size was far below required thresholds, indicating that the available evidence is insufficient to support confirmatory conclusions.

Substantial heterogeneity—arising from differences in surgical models, types, and dosages of local anesthetics, and scoring systems—also weakens the validity of the pooled results. These methodological differences likely contributed to inconsistent effect sizes and limited the generalizability of the findings, particularly given the preclinical nature of all included evidence. Moreover, the translational relevance of these animal models remains limited because species-specific healing responses, experimental surgical conditions, and dosing strategies do not fully replicate human postoperative adhesion formation. Therefore, the overall external validity of the pooled estimates remains limited, and these findings should be interpreted strictly within the context of heterogeneous preclinical models rather than as directly applicable to clinical practice.

Although different local anesthetics have distinct pharmacologic profiles, they share fundamental mechanisms related to sodium-channel blockade, nociceptive inhibition, and anti-inflammatory modulation. Therefore, pooling them as a drug class was methodologically justifiable for estimating an overall class effect; however, this approach also introduces additional variability and should be interpreted cautiously.

There was no evidence of differences in the quantity and inflammation in the conventional meta-analysis; however, the dose per coefficient significantly correlated with inflammation and fibrosis in the meta-regression analysis.

As the life expectancy increases, the likelihood of undergoing surgery also increases. Despite the development of various strategies to prevent postoperative adhesions, the overall increase in the number of surgeries has led to a higher risk of post-surgical adhesions, emphasizing the importance of addressing this issue.

Local anesthetics are used to desensitize the area surrounding the surgical site or alleviate pain. They are useful in various medical procedures, including dental work, surgery, and childbirth [40,41]. Furthermore, local anesthetics can serve as a remedy for chronic pain conditions, such as nerve pain and arthritic discomfort. They can be administered via multiple routes, including injection, topical application, and oral ingestion.

This study suggests that local anesthetics may be effective in reducing postoperative adhesions. This conclusion was supported by the sensitivity analyses, removing one study at a time and subgroup analyses across various types of local anesthetics, including lidocaine, bupivacaine, and prilocaine. Nevertheless, this should not be regarded as definitive evidence of efficacy, given the low certainty of evidence, substantial between-study heterogeneity, and the exploratory nature of several outcomes. Although there was no significant difference in the quantity and inflammation between the local anesthetics-treated and control groups, this may be due to limitations in the sample size or study power. In particular, TSA indicated that far fewer animals were included in studies analyzing adhesion quality 12.3% (104 of 845 animals), quantity (13.5%, 104 of 770 animals), and score 4.1% (119 of 2936 animals) than what would be needed to detect a difference. Importantly, these denominators correspond to the required information size estimated by trial sequential analysis rather than actual animal numbers, indicating the large sample size needed to draw confirmatory conclusions.

However, the sensitivity analysis in quantity and quality showed a statistically significant difference when the study by Suckow et al. was excluded [14]. In that study, bupivacaine was administered via a four-layer vacuum-pressed SIS material rather than being directly applied to the surgical site, and the exact dosage was not reported, possibly reducing the observed effects of local anesthetics on adhesion quality and quantity. Additionally, although no significant relationship was found between the dose per coefficient and the quality, quantity, score, or severity of adhesions, meta-regression analysis revealed a significant correlation between the dose per coefficient and both inflammation and fibrosis. This dose–response relationship between local anesthetics dose and both inflammation and fibrosis further supports the potential effectiveness of local anesthetics in reducing postoperative adhesions.

The proposed mechanisms underlying the anti-adhesive effects of local anesthetics involve both local modulation of inflammation and fibrinolysis and attenuation of the systemic surgical stress response.

Locally, tissue injury induces an inflammatory cascade that promotes fibrin deposition and adhesion formation. Local anesthetics function by blocking membrane sodium channels and temporarily stabilizing cell membranes, leading to the inhibition of nerve impulses. This prevents the release of inflammatory mediators such as substance P and calcitonin gene-related peptide (CGRP), effectively reducing the local inflammatory response [7], which has been recognized as a common cause in all pathways for adhesion formation [1]. Moreover, local anesthetics may improve tissue conditions by reducing pain-related immobility and indirectly influencing the immune response. Consistent with this mechanism, the present review identified a dose–response relationship between local anesthetics dosage and inflammation, supporting the role of local anesthetics in suppressing inflammation.

Local anesthetics also activate the fibrinolytic system, leading to reductions in factor VIII, plasminogen, and alpha-2-antiplasmin concentrations, as well as inhibition of platelet aggregation [8]. Additional mechanisms—such as reduced adhesion molecule expression after ischemia–reperfusion [27], decreased granulocyte adhesion and ROS production [28], and alterations in neovascularization and vessel-wall characteristics—may further contribute to reduced adhesion severity.

In addition to these local anti-inflammatory and fibrinolytic effects, local anesthetics may attenuate the systemic surgical stress response by reducing nociceptive afferent input to the central nervous system [42]. Blunted sympathetic activation and lower stress-related cytokine release (e.g., IL-6, TNF-α) may help prevent stress-induced tissue hypoperfusion, excessive fibrinogenesis, and delayed fibrinolysis—pathways known to promote adhesion formation. Incorporating this systemic mechanism strengthens the biological plausibility of the potential anti-adhesive properties of local anesthetics.

Compared with established anti-adhesive strategies such as hyaluronate-based barriers, NSAIDs, or corticosteroids, local anesthetics act through distinct mechanisms involving nociceptive modulation and fibrinolytic activation rather than forming a physical barrier. However, their experimental evidence base remains considerably smaller, and direct comparative studies are needed.

Postoperative adhesions predominantly occur at the surgical site, and pain is commonly experienced in the same area. While the known analgesic and anti-inflammatory properties of local anesthetics suggest their potential role as chemical barriers against adhesion formation, intraperitoneal or topical intra-abdominal administration is not a routine clinical practice. Although prior clinical studies have reported the use of intraperitoneal local anesthetics without major safety concerns [43], none of the animal studies included in this review systematically evaluated toxicity or systemic absorption. Therefore, the overall safety profile of intraperitoneal local anesthetics remains insufficiently characterized in the preclinical literature and should be carefully considered in any clinical translation.

It should also be noted that several microscopic outcomes—including severity, inflammation, and fibrosis—were derived from only one or two studies, each using different histologic grading techniques. This scarcity of data and methodological heterogeneity limits the reliability of microscopic findings, and these results should be regarded as hypothesis-generating rather than definitive evidence. Because the included studies used heterogeneous macroscopic and microscopic scoring systems, the use of standardized mean differences mitigates scale differences but cannot fully eliminate conceptual heterogeneity, which limits the interpretability of pooled effect sizes.

In addition, the methodological rigor of the included studies was limited. Several key SYRCLE domains—including random allocation, allocation concealment, random housing, and blinding—were predominantly rated as ‘not described,’ indicating substantial methodological uncertainty. This concentration of unclear judgments weakens confidence in the internal validity of the preclinical evidence and highlights the need for more rigorously designed animal studies. Furthermore, the overall certainty of evidence, assessed using a GRADE-adapted framework for preclinical research, was rated as low to very low across most outcomes due to small sample sizes, unclear bias domains, variability in scoring systems, and imprecision. Accordingly, all synthesized findings should be regarded as preliminary.

This study had several limitations. First, it had a small sample size; only six studies were included in this systematic review and meta-analysis with trial sequential analysis, and no outcomes in this study reached the RIS; thus, it may have been underpowered. Secondly, the meta-analysis revealed substantial heterogeneity in some outcomes. The included studies were conducted under diverse protocols with varying types and concentrations of local anesthetics and different types of surgery, which can lead to considerable heterogeneity. We conducted a subgroup analysis according to the type of local anesthetics and performed sensitivity and meta-regression analyses of all included outcomes. Thirdly, none of the included studies systematically assessed toxicity, so the safety profile of local anesthetics in preventing postoperative adhesions remains uncertain. Although several clinical trials have previously administered intraperitoneal local anesthetics for postoperative pain management without reporting major safety concerns, these studies were designed for analgesia rather than adhesion prevention, and their findings cannot substitute for a dedicated evaluation of toxicity in the context of anti-adhesive use. Finally, as the included studies were experimental, more recent evidence from human trials on local anesthetics is required for clinical application. As evidence of a preclinical investigation, the findings of our study can serve as a basis for clinical trials.

Despite these constraints, our research demonstrated robustness by employing a stringent methodology to conduct an inaugural systematic review and meta-analysis with trial sequential analysis to assess the anti-adhesive effects of local anesthetics in preventing postoperative adhesions.

Future research should employ standardized adhesion models, unified scoring systems, and systematic toxicity assessments. Comparative experiments on different types and doses of local anesthetics, and administration timing are required to determine which agents offer the most favorable anti-adhesive profile. Greater methodological harmonization will be essential for improving reproducibility and strengthening translational relevance.

## 5. Conclusions

Local anesthetics showed a potential to reduce postoperative adhesion formation in preclinical models; however, the overall certainty of evidence was low to very low due to small sample sizes, substantial methodological heterogeneity, and a high proportion of unclear risk-of-bias domains. TSA further demonstrated that the accumulated information size was insufficient to draw confirmatory conclusions. Future studies using standardized adhesion models, rigorous bias control, and systematic safety assessments are required before any implications for clinical practice can be established.

## Figures and Tables

**Table 1:** Characteristics of included studies.

First Author, Publication Year	Animal	Surgery	Group	Definition
Yuzbasioglu, 2008 [15]	Wistar rats	Laparotomy	Control	No irrigation
			Group 1	Lidocaine 2.5%, prilocaine 2.5%
			Group 2	Lidocaine 2.5% (1 mg/kg)
			Group 3	Ceftriaxone (100 mg/kg)
Ozturk, 2010 [17]	Wistar-Albino rats	Laparotomy	Control	No irrigation
			Group 1	Saline (5 mL)
			Group 2	Lidocaine (7 mg/kg)
			Group 3	Lidocaine (3 mg/kg)
			Group 4	Bupivacaine (2 mg/kg)
Suckow, 2012 [14]	Sprague Dawley rats	Laparotomy	Group 1	No irrigation
			Group 2	Bupivacaine
Parsa, 2017 [16]	Sprague Dawley rats	Laparotomy	Control	No irrigation
			Group 2	Normal saline (0.9% sodium chloride solution)
			Group 3	Lidocaine 2% (3 mg/kg)
			Group 4	Lidocaine 2% (6 mg/kg)
			Group 5	Bupivacaine
Choi, 2017 [18]	Sprague Dawley rats	Incisional pain model	Group S	No irrigation
			Group P	PACM
			Group PL0.5	0.5% lidocaine-loaded PACM
			Group PL1	1% lidocaine-loaded PACM
			Group PL2	2% lidocaine-loaded PACM
			Group PL4	4% lidocaine-loaded PACM
Kesici, 2024 [13]	Sprague Dawley rats	Laparotomy + colon anastomosis	Group C	Isotonic solution
			Group B	Pre-incisional bupivacaine 2 mg/kg + peritoneal bupivacaine 2 mg/kg
			Group L	Pre-incisional levobupivacaine 2.5 mg/kg + peritoneal levobupivacaine 2.5 mg/kg

**Table 2:** Summary of the meta-analysis.

	Outcome	No. of Studies	No. of Animals	Conventional Meta-Analysis		Trial Sequential Analysis		
				SMD with 95% CI	Heterogeneity (I^2^)	Conventional Test Boundary	Monitoring Boundary	RIS
Macroscopic adhesion score	Quality (or tenacity of adhesion)	3	104	Significant (SMD: −0.996; 95% CI −1.906 to −0.085)	72.6	Cross	Not cross	12.3% (104 of 845 animals)
	Quantity (or extent of adhesion)	3	104	Not significant (SMD −0.544; 95% CI −1.452 to 0.365)	77.6	Not cross	Not cross	13.5% (104 of 770 animals)
	Total adhesion score	4	98	Significant (SMD −1.528; 95% CI −2.081 to −0.976)	30.0	Cross	Not cross	4.1% (119 of 2936 animals)
Microscopic adhesion score	Severity	2	80	Significant (SMD −1.304; 95% CI −1.862 to −0.746)	31.7			
	Inflammation	1	84	Not significant (SMD: −1.833; 95% CI −4.017 to 0.350)	90.9			
	Fibrosis	1	84	Significant (SMD: −2.373; 95% CI −3.400 to −1.346)	60.4			

No., number; SMD, standardized mean difference; CI, confidence interval; RIS, required information size.

## Data Availability

All relevant data are included in the manuscript and the Supplementary Data within the Supplementary Information files.

## Supplementary Materials

The following supporting information can be downloaded at: https://www.mdpi.com/article/10.3390/medicina61122215/s1.

## Abbreviations

The following abbreviations are used in this manuscript:

No	number
LA	local anesthetic
SMD	standardized mean difference
MD	mean difference
CI	confidence interval
TSA	trial sequential analysis
RIS	required information size
