# Supplementary material for: Effect of Local Anesthetics on Experimental Postoperative Adhesion: A Systematic Review and Meta-Analysis with Trial Sequential Analysis
Source: Medicina (Kaunas). 2025 Dec 15;61(12):2215. doi: 10.3390/medicina61122215 (PMC12734440; doi:10.3390/medicina61122215)
Supplement: Supplementary file 1 [file medicina-61-02215-s001.zip › medicina-4007396-Supplementary.pdf]

Supplementary Information

Search strategy

Ovid-Medline

SD	1	Exp General Surgery	440470
	2	surgery.mp.	2817407
	3	operation.mp	23546
	4	Opera*.mp	1217674
	5	Surg*.mp	3282981
	6	or/1-5	3852560
	7.	exp Tissue Adhesions/	13340
	8	adhesion.mp	274714
	9	adhesive.mp	54193
	10	or/7-9	318751
	11	6 and 10	36625
	12	Exp anesthetics, local/	110759
	13	local anesthetic.mp	11788
	14	local anaesthetic.mp	5516
	15	Bupivacaine.mp	15975
	16	Chloroprocaine.mp	493
	17	Cinchocaine.mp	105
	18	levobupivacaine.mp	1321
	19	lidocaine.mp	32484
	20	lignocaine.mp	2672
	21	mepivacaine.mp	2648
	22	Prilocaine.mp	2650
	23	procaine.mp	13333
	24	ropivacaine.mp	4982
	25	Tetracaine.mp	3347
	26	xylocaine.mp	1042
	27	or/12-26	127879
	28	11 and 27	175

Embase

SD	1	**'general surgery'**/exp	180,218
	2	**surgery:ti,ab**	1,929,529
	3	**operation:ti,ab**	516,331
	4	Surg*:ti,ab	2,036,873
	5	Operat*:ti,ab	653534
	6	#1 OR #2 OR #3 OR #4 OR #5	2,434,850
	7.	#1 OR #2 OR #3 OR #4 OR #5 AND [embase]/lim	1,991,646
	8	'adhesion'/exp	64,447
	9	Adhesion:ti,ab	277,050
	10	'adhesion barrier'/exp	715
	11	#8 OR #9 OR #10	312,191
	12	#7 AND #11	18,279
	13	'local anesthetic agent'/exp	289,700
	14	local AND anesthetic**:ti,ab**	24,403
	15	local AND anaesthetic**:ti,ab**	13,697
	16	Bupivacaine**:ti,ab**	20,863
	17	Chloroprocaine**:ti,ab**	616
	18	Cinchocaine**:ti,ab**	142
	19	Prilocaine.	7,900
	20	Tetracaine	7,714
	21	lidocaine**:ti,ab**	32,761
	22	lignocaine**:ti,ab**	4,036
	23	mepivacaine**:ti,ab**	2,300
	24	ropivacaine**:ti,ab**	8,331
	25	levobupivacaine**:ti,ab**	2,730
	26	procaine**:ti,ab**	6,166
	27	xylocaine**:ti,ab**	1,498

	28	#13 OR #14 OR #15 OR #16 OR #17 OR #18 OR #19 OR #20 OR #21 OR #22 OR #23 OR #24 OR #25 OR #26 OR #27	306,596
	29	#12 AND #28	195

ID	Search	Hits
#1	MeSH descriptor: [General Surgery] explode all trees	452
#2	operation	47287
#3	opera*	130562
#4	surg*	333988
#5	surgery	289014
#6	#1 or #2 or #3 or #4 or #5	375438
#7	MeSH descriptor: [Tissue Adhesives] explode all trees	575
#8	adhesion	7313
#9	adhesive	6671
#10	#7 or #8 or #9	13681
#11	#6 and #10	5323
#12	MeSH descriptor: [Anesthetics, Local] explode all trees	9727
#13	local anesthetic	13691
#14	local anaesthetic	13691
#15	Bupivacaine	15322
#16	Chloroprocaine	326
#17	Cinchocaine	33
#18	levobupivacaine	2247
#19	lidocaine	15026
#20	lignocaine	2372
#21	mepivacaine	1078
#22	Prilocaine	1456
#23	procaine	682
#24	ropivacaine	7981
#25	Tetracaine	755
#26	xylocaine	469
#27	#12 or #13 or #14 or #15 or #16 or #17 or #18 or #19 or #20 or #21 or #22 or #23 or #24 or #25 or #26	43258
#28	#11 and #27	202

Supplementary Data

Score

Study Name	Type and Dose	C-Mean	C-SD	C-N	LA-Mean	LA-SD	LA-N
Parsa,2017_1	Lidocaine 3mg/kg	5.14	1.07	3	3.3	1.34	10
Parsa,2017_2	Lidocaine 6mg/kg	5.14	1.07	3	1.2	1.79	10
Parsa,2017_3	Bupivacaine 2 mg/kg	5.14	1.07	4	1.75	1.28	10
Oztruk, 2010_1	Prilocaine 7 mg/kg	7	3.7	3	5	2.96	10
Oztruk, 2010_2	Lidocaine 3 mg/kg	7	3.7	3	6	2.22	10
Oztruk, 2010_3	Bupivacaine 2 mg/kg	7	3.7	4	4.5	2.22	10
Yuzbasioglu, 2008_1	EMLA1g	7.1	1.22	3	4.31	2.11	6
Yuzbasioglu, 2008_2	Lidocaine 1mg/kg	7.1	1.22	3	5.55	1.45	6
Kesici, 2024_1	Bupivacaine 2 mg/kg	1	0.01	3	0.14	0.38	7
Kesici, 2024_2	Levobupivacaine 2.5 mg/kg	1	0.01	4	0.14	0.38	7

C; control group, LA; local anesthetic group, SD; standard deviation, N; number of animals

Quality

Study Name	Type and Dose	C-Mean	C-SD	C-N	LA-Mean	LA-SD	LA-N
Suckow,2011	Bupivacaine	1.42	1.42	12	3.25	3.25	12
Parsa,2017	Lidocaine 3mg/kg	2.43	0.53	3	1.5	0.84	10
Parsa,2017	Lidocaine 6mg/kg	2.43	0.53	3	0.6	0.89	10
Parsa,2017	Bupivacaine 2 mg/kg	2.43	0.53	4	0.63	0.74	10
Oztruk, 2010	Prilocaine 7 mg/kg	3	1.48	3	2	1.48	10
Oztruk, 2010	Lidocaine 3 mg/kg	3	1.48	3	2	1.48	10
Oztruk, 2010	Bupivacaine 2 mg/kg	3	1.48	4	2	0.74	10

C; control group, LA; local anesthetic group, SD; standard deviation, N; number of animals

Quantity

Study Name	Type and Dose	C-Mean	C-SD	C-N	LA-Mean	LA-SD	LA-N
Suckow,2011	Bupivacaine	1.33	1.18	12	2.75	1.3	12
Parsa,2017	Lidocaine 3mg/kg	2.14	0.69	3	1.4	0.7	10
Parsa,2017	Lidocaine 6mg/kg	2.14	0.69	3	0.6	0.89	10
Parsa,2017	Bupivacaine 2 mg/kg	2.14	0.69	4	0.63	0.74	10
Oztruk, 2010	Prilocaine 7 mg/kg	2	1.48	3	2	1.48	10
Oztruk, 2010	Lidocaine 3 mg/kg	2	1.48	3	2	0.74	10
Oztruk, 2010	Bupivacaine 2 mg/kg	2	1.48	4	1.5	0.74	10

C; control group, LA; local anesthetic group, SD; standard deviation, N; number of animals

Severity

Study Name	Type and Dose	C-Mean	C-SD	C-N	LA-Mean	LA-SD	LA-N
Parsa,2017	Lidocaine 3mg/kg	2.67	0.51	3	0.5	0.84	10
Parsa,2017	Lidocaine 6mg/kg	2.67	0.51	3	0.8	1.1	10
Parsa,2017	Bupivacaine	2.67	0.51	4	1.25	1.28	10
Oztruk, 2010	Prilocaine 7 mg/kg	2	0.74	3	1	0.74	10
Oztruk, 2010	Lidocaine 3 mg/kg	2	0.74	3	2	1.48	10
Oztruk, 2010	Bupivacaine 2 mg/kg	2	0.74	4	1	0.74	10

C; control group, LA; local anesthetic group, SD; standard deviation, N; number of animals

Inflammation

Study Name	Type and Dose	C-Mean	C-SD	C-N	LA-Mean	LA-SD	LA-N
Choi,2017_1	Lidocaine 0.5%	2	0.01	3	2	0.258199	12
Choi,2017_1	Lidocaine 1%	2	0.01	3	2.333333	0.333333	12
Choi,2017_1	Lidocaine 2%	2	0.01	3	1.333333	0.210819	12
Choi,2017_1	Lidocaine 4%	2	0.01	3	1.666667	0.210819	12
Choi,2017_2	Lidocaine 4%	2.5	0.223607	12	1.333	0.2108	12

C; control group, LA; local anesthetic group, SD; standard deviation, N; number of animals

Fibrosis

Study Name	Type and Dose	C-Mean	C-SD	C-N	LA-Mean	LA-SD	LA-N
Choi,2017_1	Lidocaine 0.5%	3.166667	0.166667	3	3	0.1	12
Choi,2017_1	Lidocaine 1%	3.166667	0.166667	3	2.833333	0.307318	12
Choi,2017_1	Lidocaine 2%	3.166667	0.166667	3	2.666667	0.210819	12
Choi,2017_1	Lidocaine 4%	3.166667	0.166667	3	2.5	0.223607	12
Choi,2017_2	Lidocaine 4%	3.833333	0.166667	12	3	0.2582	12

C; control group, LA; local anesthetic group, SD; standard deviation, N; number of animals

Supplementary Figures

Supplementary Figure S1. Forest plot showing sensitivity analysis with removing one study at a time for the effect of local anesthetics on quality of adhesion compared with control. The figure depicts individual trials as filled squares with relative sample size and the 95% confidence interval (CI) of the difference as a solid line. The diamond shape indicates the pooled estimate and uncertainty for the combined effect.

Study name	Subgroup within study	Point	Lower limit	Upper limit
Suckow,2011	Bupivacaine	-1.307	-1.922	-0.691
Parsa,2017	Bupivacaine 2 mg/kg	-0.735	-1.602	0.133
Parsa,2017	Lidocaine 3 mg/kg	-0.981	-2.032	0.070
Parsa,2017	Lidocaine 6 mg/kg	-0.815	-1.757	0.128
Oztruk, 2010	Bupivacaine 2 mg/kg	-1.009	-2.086	0.068
Oztruk, 2010	Lidocaine 3 mg/kg	-1.066	-2.138	0.006
Oztruk, 2010	Prilocaine 7 mg/kg	-1.066	-2.138	0.006
		-0.996	-1.906	-0.085

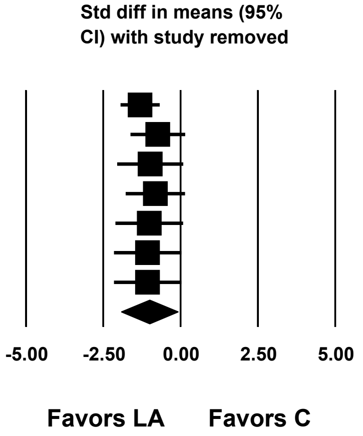

Supplementary Figure S2. Forest plot showing effect of lidocaine on quality of adhesion compared with control. The figure depicts individual trials as filled squares with relative sample size and the 95% confidence interval (CI) of the difference as a solid line. The diamond shape indicates the pooled estimate and uncertainty for the combined effect.

Study name	Statistics for each study		
	Std diff in means	Lower limit	Upper limit
Parsa,2017_1	-1.227	-2.386	-0.067
Parsa,2017_2	-2.297	-3.649	-0.945
Oztruk, 2010	-0.676	-1.577	0.226
	-1.190	-1.819	-0.560

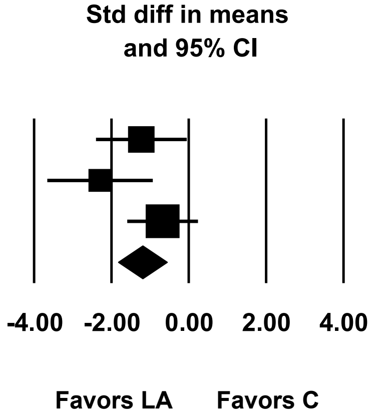

Supplementary Figure S3. Forest plot showing effect of bupivacaine on quality of adhesion compared with control. The figure depicts individual trials as filled squares with relative sample size and the 95% confidence interval (CI) of the difference as a solid line. The diamond shape indicates the pooled estimate and uncertainty for the combined effect.

Study name	Statistics for each study		
	Std diff in means	Lower limit	Upper limit
Suckow,2011	0.730	-0.097	1.556
Parsa,2017	-2.797	-4.029	-1.564
Oztruk, 2010	-0.855	-1.770	0.061
	-0.928	-2.814	0.959

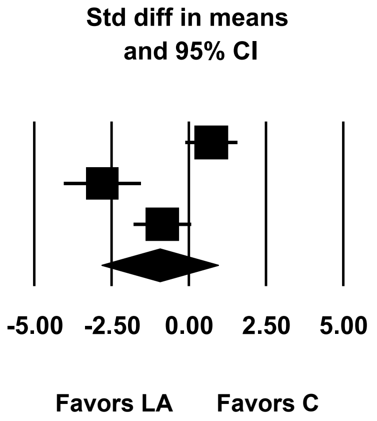

Supplementary Figure S4. Forest plot showing effect of prilocaine on quality of adhesion compared with control. The figure depicts individual trials as filled squares with relative sample size and the 95% confidence interval (CI) of the difference as a solid line. The diamond shape indicates the pooled estimate and uncertainty for the combined effect.

Study name	Statistics for each study			Std diff in means and 95% CI
	Std diff in means	Lower limit	Upper limit	
Oztruk, 2010	-0.676	-1.577	0.226	
	-0.676	-1.577	0.226	

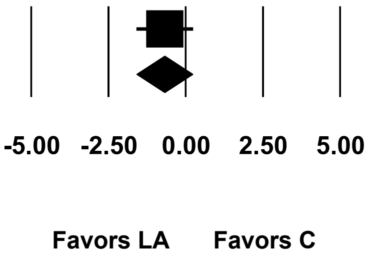

Supplementary Figure S5. Meta-regression scatter plot showing standardized difference in means of quality against dose per coefficient. The figure depicts individual trials as hollow circle. The size of the circle is drawn proportional to the weight that the studies.

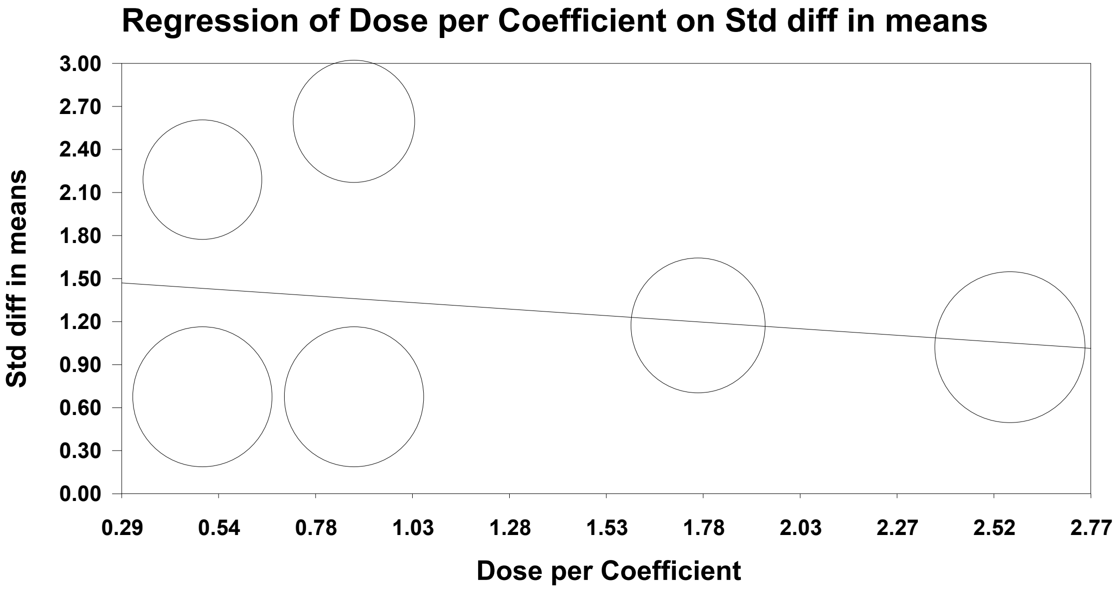

Supplementary Figure S6. Forest plot showing effect of local anesthetics on quantity of adhesion compared with control. The figure depicts individual trials as filled squares with relative sample size and the 95% confidence interval (CI) of the difference as a solid line. The diamond shape indicates the pooled estimate and uncertainty for the combined effect.

Study name	Subgroup within study	Statistics for each study		
		Std diff in means	Lower limit	Upper limit
Suckow,2011	Bupivacaine	1.144	0.281	2.007
Parsa,2017	Bupivacaine 2 mg/kg	-2.075	-3.466	-0.684
Parsa,2017	Lidocaine 3 mg/kg	-1.060	-2.413	0.293
Parsa,2017	Lidocaine 6 mg/kg	-1.797	-3.260	-0.333
Oztruk, 2010	Bupivacaine 2 mg/kg	-0.511	-1.686	0.664
Oztruk, 2010	Lidocaine 3 mg/kg	0.000	-1.290	1.290
Oztruk, 2010	Prilocaine 7 mg/kg	0.000	-1.290	1.290
		-0.544	-1.452	0.365

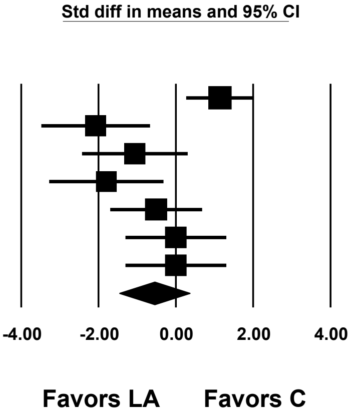

Supplementary Figure S7. Forest plot showing sensitivity analysis with removing one study at a time for the effect of local anesthetics on quantity of adhesion compared with control. The figure depicts individual trials as filled squares with relative sample size and the 95% confidence interval (CI) of the difference as a solid line. The diamond shape indicates the pooled estimate and uncertainty for the combined effect.

Study name	Subgroup within study	Point	Lower limit	Upper limit
Suckow,2011	Bupivacaine	-0.858	-1.555	-0.162
Parsa,2017	Bupivacaine 2 mg/kg	-0.288	-1.163	0.587
Parsa,2017	Lidocaine 3 mg/kg	-0.469	-1.496	0.557
Parsa,2017	Lidocaine 6 mg/kg	-0.350	-1.288	0.587
Oztruk, 2010	Bupivacaine 2 mg/kg	-0.564	-1.646	0.518
Oztruk, 2010	Lidocaine 3 mg/kg	-0.646	-1.710	0.418
Oztruk, 2010	Prilocaine 7 mg/kg	-0.646	-1.710	0.418
		-0.544	-1.452	0.365

Std diff in means (95% CI) with study removed

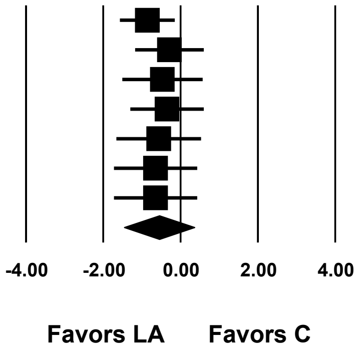

Supplementary Figure S8. Forest plot showing effect of bupivacaine on quantity of adhesion compared with control. The figure depicts individual trials as filled squares with relative sample size and the 95% confidence interval (CI) of the difference as a solid line. The diamond shape indicates the pooled estimate and uncertainty for the combined effect.

Study name	Statistics for each study		
	Std diff in means	Lower limit	Upper limit
Suckow,2011	1.144	0.281	2.007
Parsa,2017	-2.075	-3.466	-0.684
Oztruk, 2010	-0.511	-1.686	0.664
	-0.418	-2.261	1.426

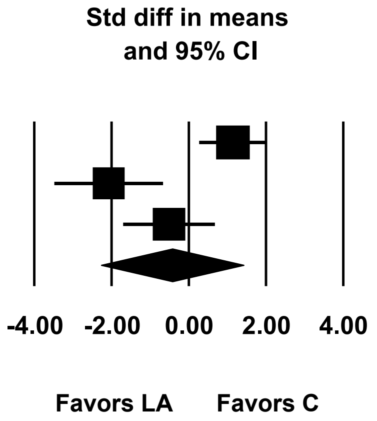

Supplementary Figure S9. Forest plot showing effect of lidocaine on quantity of adhesion compared with control. The figure depicts individual trials as filled squares with relative sample size and the 95% confidence interval (CI) of the difference as a solid line. The diamond shape indicates the pooled estimate and uncertainty for the combined effect.

Study name	Statistics for each study		
	Std diff in means	Lower limit	Upper limit
Parsa,2017_1	-1.062	-2.201	0.077
Parsa,2017_2	-1.847	-3.108	-0.587
Oztruk, 2010	0.000	-0.877	0.877
	-0.895	-1.980	0.189

Std diff in means and 95% CI

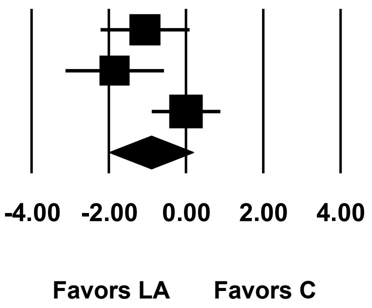

Supplementary Figure S10. Forest plot showing effect of prilocaine on quantity of adhesion compared with control. The figure depicts individual trials as filled squares with relative sample size and the 95% confidence interval (CI) of the difference as a solid line. The diamond shape indicates the pooled estimate and uncertainty for the combined effect.

Study name	Statistics for each study			Std diff in means and 95% CI
	Std diff in means	Lower limit	Upper limit	
Oztruk, 2010	0.000	-0.877	0.877	
	0.000	-0.877	0.877	

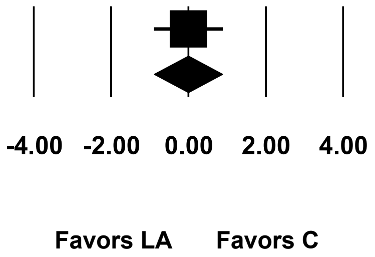

Supplementary Figure S11. Meta-regression scatter plot showing standardized difference in means of quantity against dose per coefficient. The figure depicts individual trials as hollow circle. The size of the circle is drawn proportional to the weight that the studies.

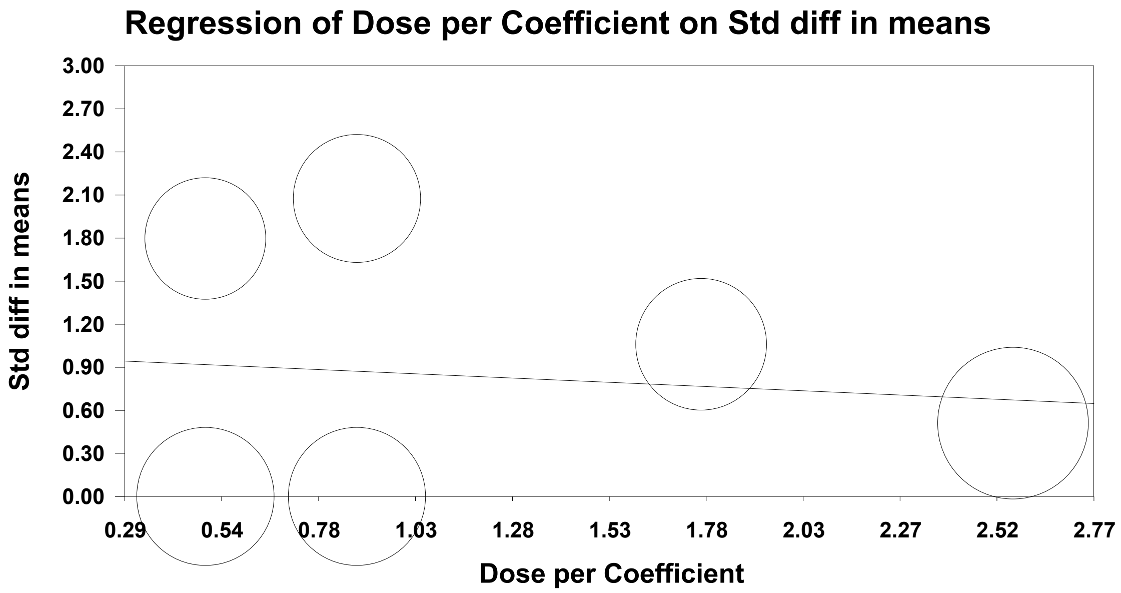

Supplementary Figure S12. Trial sequential analysis showing effect of local anesthetics on quantity of adhesion compared with control. The trial sequential analysis (TSA) for the studies comparing the effect of local anesthetics on quality of adhesion with control.The uppermost and lowermost curves represent trial sequential monitoring boundary lines for benefit and harm, respectively. The horizontal line represents the conventional boundaries for statistical significance. The triangular lines on the right side reflect the futility boundaries. The number on the x-axis indicates the required information size.

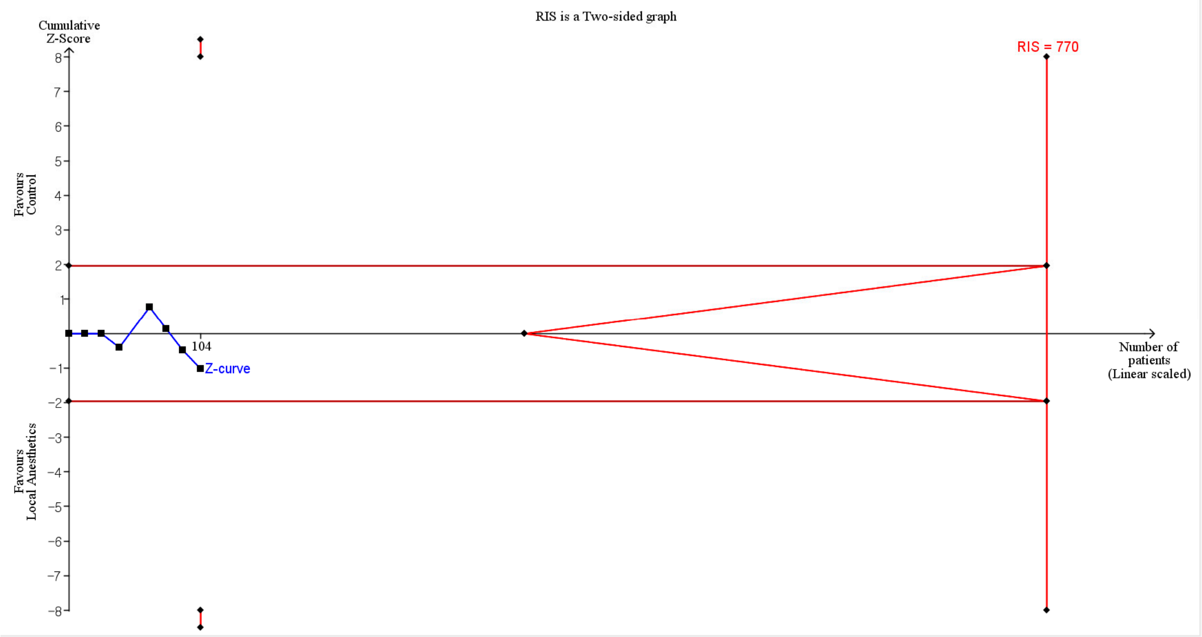

Supplementary Figure S13. Forest plot showing sensitivity analysis with removing one study at a time for the effect of local anesthetics on score of adhesion compared with control. The figure depicts individual trials as filled squares with relative sample size and the 95% confidence interval (CI) of the difference as a solid line. The diamond shape indicates the pooled estimate and uncertainty for the combined effect

Study name	Subgroup within study	Point	Lower limit	Upper limit
Parsa,2017	Bupivacaine 2 mg/kg	-1.386	-1.927	-0.846
Parsa,2017	Lidocaine 3mg/kg	-1.556	-2.177	-0.934
Parsa,2017	Lidocaine 6mg/kg	-1.449	-2.031	-0.866
Oztruk, 2010	Bupivacaine 2 mg/kg	-1.622	-2.232	-1.012
Oztruk, 2010	Lidocaine 3 mg/kg	-1.663	-2.209	-1.117
Oztruk, 2010	Prilocaine 7 mg/kg	-1.642	-2.223	-1.061
Yuzbasioglu, 2008	EMLA1g	-1.548	-2.163	-0.933
Yuzbasioglu, 2008	Lidocaine 1mg/kg	-1.585	-2.197	-0.974
Kesici, 2024	Bupivacaine 2 mg/kg	-1.437	-2.002	-0.871
Kesici, 2024	Levobupivacaine 2.5 mg/kg	-1.408	-1.956	-0.860
		-1.528	-2.081	-0.976

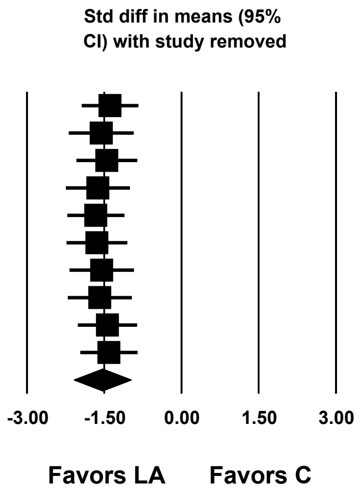

Supplementary Figure S14. Forest plot showing effect of lidocaine on score of adhesion compared with control. The figure depicts individual trials as filled squares with relative sample size and the 95% confidence interval (CI) of the difference as a solid line. The diamond shape indicates the pooled estimate and uncertainty for the combined effect.

Study name	Statistics for each study		
	Std diff in means	Lower limit	Upper limit
Parsa,2017_1	-1.457	-2.650	-0.263
Parsa,2017_2	-2.457	-3.845	-1.070
Oztruk, 2010	-0.328	-1.210	0.555
Yuzbasioglu, 2008	-1.186	-2.278	-0.094
	-1.252	-2.107	-0.397

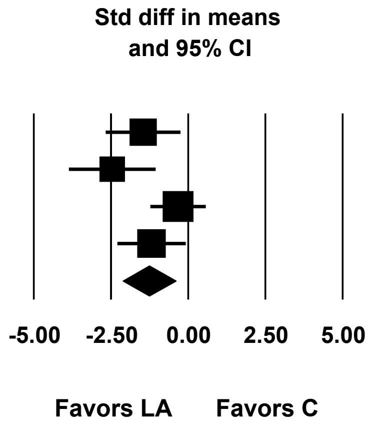

Supplementary Figure S15. Forest plot showing effect of bupivacine on score of adhesion compared with control. The figure depicts individual trials as filled squares with relative sample size and the 95% confidence interval (CI) of the difference as a solid line. The diamond shape indicates the pooled estimate and uncertainty for the combined effect.

Study name	Statistics for each study		
	Std diff in means	Lower limit	Upper limit
Parsa,2017	-2.874	-4.123	-1.624
Oztruk, 2010	-0.819	-1.732	0.093
Kesici, 2024	-2.613	-4.385	-0.841
	-2.010	-3.480	-0.541

Std diff in means and 95% CI

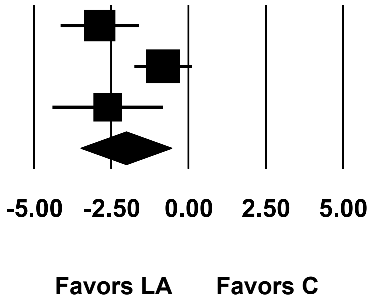

Supplementary Figure S16. Forest plot showing effect of bupivacine on score of adhesion compared with control. The figure depicts individual trials as filled squares with relative sample size and the 95% confidence interval (CI) of the difference as a solid line. The diamond shape indicates the pooled estimate and uncertainty for the combined effect.

Study name	Statistics for each study			Std diff in means and 95% CI
	Std diff in means	Lower limit	Upper limit	
Kesici, 2024	-2.613	-4.385	-0.841	
	-2.613	-4.385	-0.841	

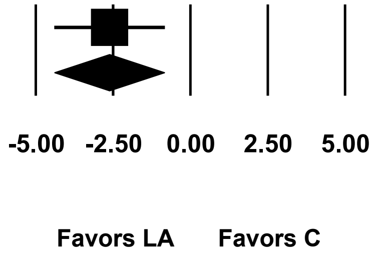

Supplementary Figure S17. Forest plot showing effect of EMLA on score of adhesion compared with control. The figure depicts individual trials as filled squares with relative sample size and the 95% confidence interval (CI) of the difference as a solid line. The diamond shape indicates the pooled estimate and uncertainty for the combined effect.

Study name	Statistics for each study		
	Std diff in means	Lower limit	Upper limit
Yuzbasioglu, 2008	-1.619	-2.923	-0.315
	-1.619	-2.923	-0.315

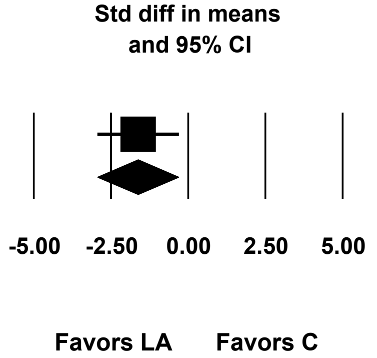

Supplementary Figure S18. Forest plot showing effect of prilocaine on score of adhesion compared with control. The figure depicts individual trials as filled squares with relative sample size and the 95% confidence interval (CI) of the difference as a solid line. The diamond shape indicates the pooled estimate and uncertainty for the combined effect.

Study name	Statistics for each study			Std diff in means and 95% CI
	Std diff in means	Lower limit	Upper limit	
Oztruk, 2010	-0.597	-1.493	0.299	
	-0.597	-1.493	0.299	

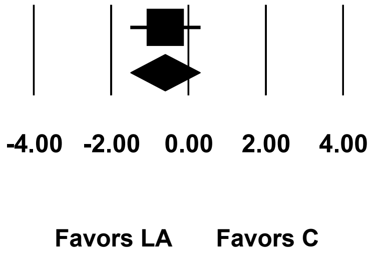

Supplementary Figure S19. Meta-regression scatter plot showing standardized difference in means of score against dose per coefficient. The figure depicts individual trials as hollow circle. The size of the circle is drawn proportional to the weight that the studies.

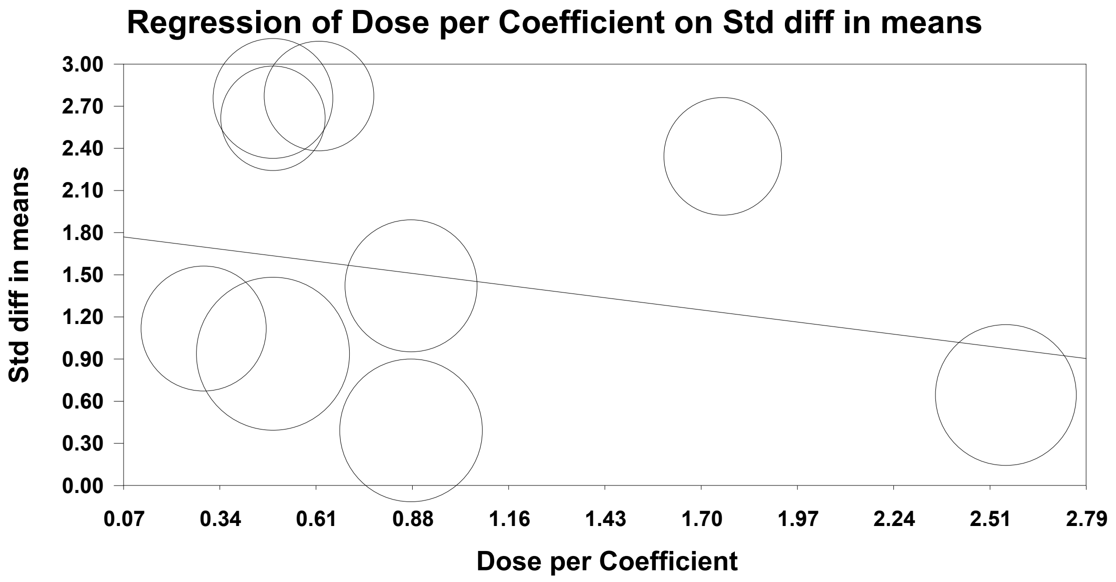

Supplementary Figure S20. Forest plot showing sensitivity analysis with removing one study at a time for the effect of local anesthetics on severity of adhesion compared with control. The figure depicts individual trials as filled squares with relative sample size and the 95% confidence interval (CI) of the difference as a solid line. The diamond shape indicates the pooled estimate and uncertainty for the combined effect

Study name	Subgroup within study	Point	Lower limit	Upper limit
Parsa,2017	Bupivacaine	-1.318	-1.942	-0.694
Parsa,2017	Lidocaine 3mg/kg	-1.122	-1.714	-0.529
Parsa,2017	Lidocaine 6mg/kg	-1.214	-1.818	-0.611
Oztruk, 2010	Bupivacaine 2 mg/kg	-1.293	-1.915	-0.670
Oztruk, 2010	Lidocaine 3 mg/kg	-1.604	-2.224	-0.985
Oztruk, 2010	Prilocaine 7 mg/kg	-1.295	-1.904	-0.685
		-1.304	-1.862	-0.746

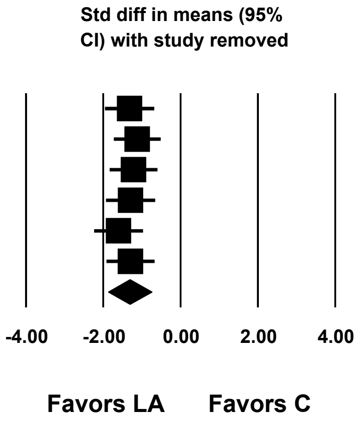

Supplementary Figure S21. Forest plot showing effect of bupivacaine on severity of adhesion compared with control. The figure depicts individual trials as filled squares with relative sample size and the 95% confidence interval (CI) of the difference as a solid line. The diamond shape indicates the pooled estimate and uncertainty for the combined effect.

Study name	Statistics for each study		
	Std diff in means	Lower limit	Upper limit
Parsa,2017	-1.457	-2.444	-0.471
Oztruk, 2010	-1.351	-2.323	-0.380
	-1.404	-2.096	-0.712

Std diff in means and 95% CI

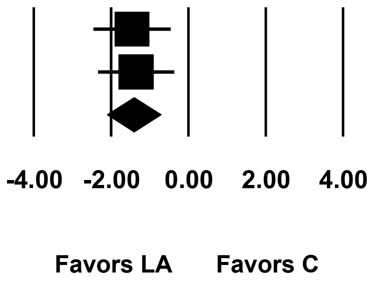

Supplementary Figure S22. Forest plot showing effect of prilocaine on severity of adhesion compared with control. The figure depicts individual trials as filled squares with relative sample size and the 95% confidence interval (CI) of the difference as a solid line. The diamond shape indicates the pooled estimate and uncertainty for the combined effect.

Study name	Statistics for each study			Std diff in means and 95% CI
	Std diff in means	Lower limit	Upper limit	
Oztruk, 2010	-1.351	-2.323	-0.380	
	-1.351	-2.323	-0.380	

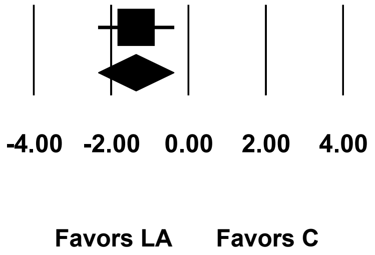

Supplementary Figure S23. Forest plot showing effect of lidocaine on severity of adhesion compared with control. The figure depicts individual trials as filled squares with relative sample size and the 95% confidence interval (CI) of the difference as a solid line. The diamond shape indicates the pooled estimate and uncertainty for the combined effect.

Study name	Statistics for each study		
	Std diff in means	Lower limit	Upper limit
Parsa,2017_1	-2.878	-4.366	-1.390
Parsa,2017_2	-1.952	-3.233	-0.671
Oztruk, 2010	0.000	-0.877	0.877
	-1.534	-3.314	0.246

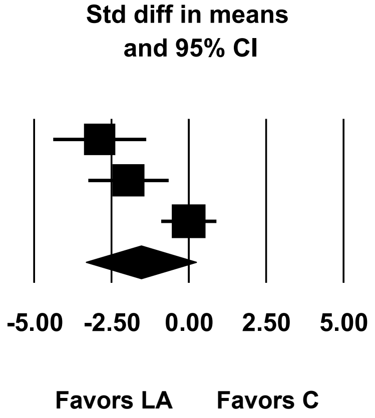

Supplementary Figure S24. Meta-regression scatter plot showing standardized difference in means of severity against dose per coefficient. The figure depicts individual trials as hollow circle. The size of the circle is drawn proportional to the weight that the studies.

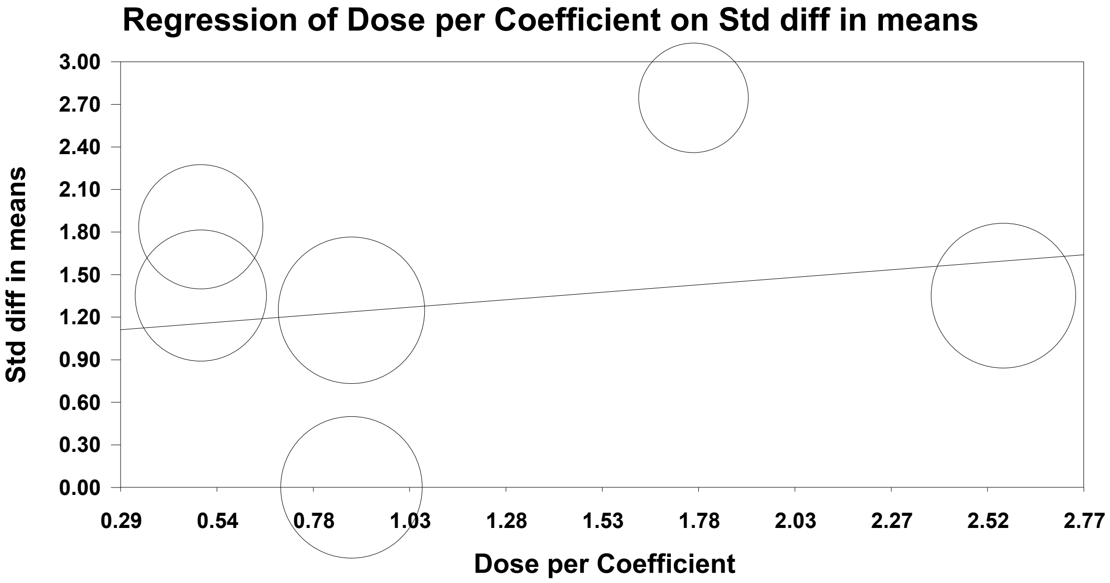

Supplementary Figure S25. Forest plot showing effect of local anesthetics on inflammation compared with control. The figure depicts individual trials as filled squares with relative sample size and the 95% confidence interval (CI) of the difference as a solid line. The diamond shape indicates the pooled estimate and uncertainty for the combined effect.

Study name	Subgroup within study	Statistics for each study		
		Std diff in means	Lower limit	Upper limit
Choi,2017_1	Lidocaine 0.5%	0.000	-1.265	1.265
Choi,2017_1	Lidocaine 1%	1.087	-0.237	2.411
Choi,2017_1	Lidocaine 2%	-3.437	-5.202	-1.673
Choi,2017_1	Lidocaine 4%	-1.719	-3.125	-0.312
Choi,2017_2	Lidocaine 4%	-5.371	-7.088	-3.653
		-1.833	-4.017	0.350

Std diff in means and 95% CI

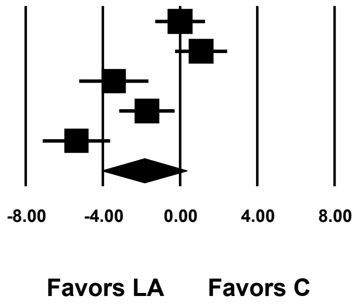

Supplementary Figure S26. Meta-regression scatter plot showing standardized difference in means of inflammation against dose per coefficient. The figure depicts individual trials as hollow circle. The size of the circle is drawn proportional to the weight that the studies.

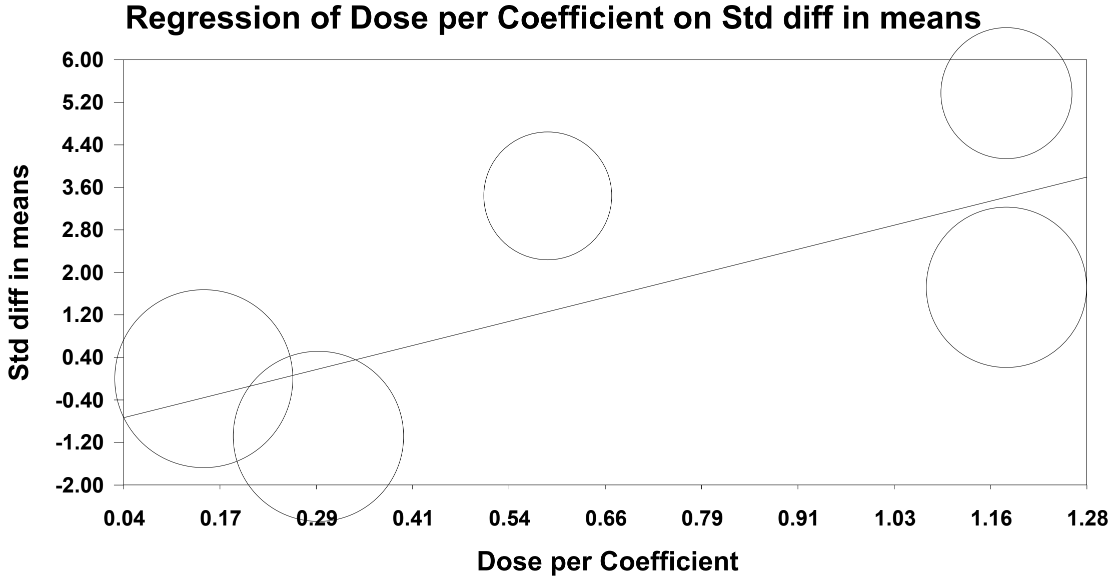

Supplementary Figure S27. Forest plot showing effect of local anesthetics on fibrosis compared with control. The figure depicts individual trials as filled squares with relative sample size and the 95% confidence interval (CI) of the difference as a solid line. The diamond shape indicates the pooled estimate and uncertainty for the combined effect.

Study name	Subgroup within study	Statistics for each study		
		Std diff in means	Lower limit	Upper limit
Choi,2017_1	Lidocaine 0.5%	-1.477	-2.848	-0.106
Choi,2017_1	Lidocaine 1%	-1.149	-2.479	0.181
Choi,2017_1	Lidocaine 2%	-2.443	-3.981	-0.905
Choi,2017_1	Lidocaine 4%	-3.089	-4.769	-1.409
Choi,2017_2	Lidocaine 4%	-3.835	-5.183	-2.487
		-2.373	-3.400	-1.346

Std diff in means and 95% CI

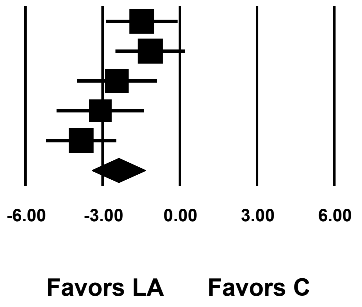

Supplementary Figure S28. Meta-regression scatter plot showing standardized difference in means of fibrosis against dose per coefficient. The figure depicts individual trials as hollow circle. The size of the circle is drawn proportional to the weight that the studies.

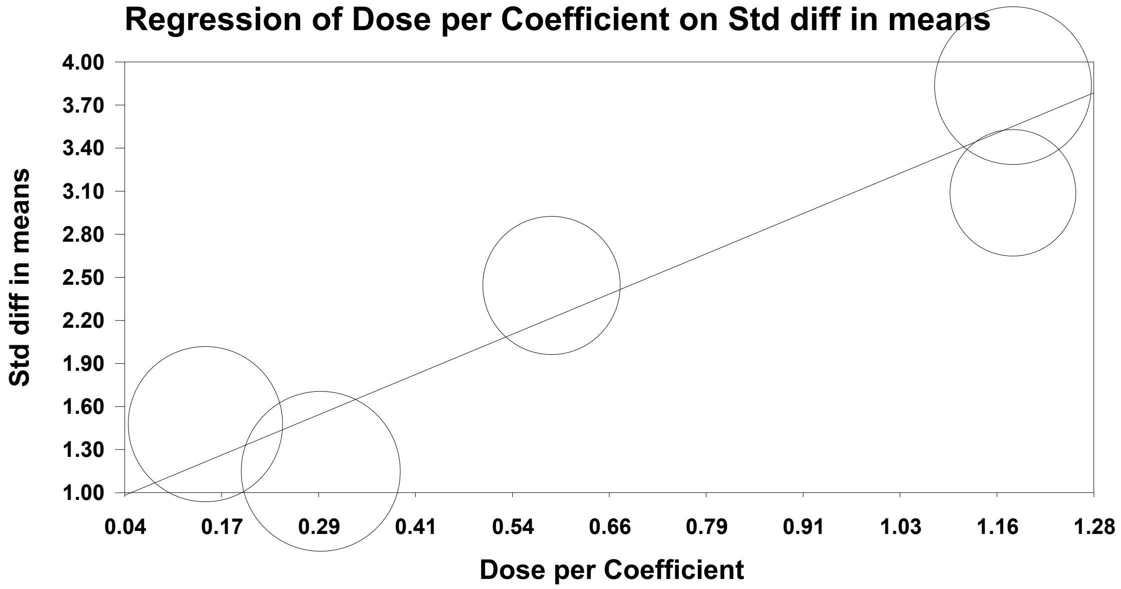

Supplementary Table S1. Weights for each study for pooled analysis

		Author,Year	Kinds and dose of local anestheics	Weight(%)
Quality	Total	Suckow,2011	Bupivacaine	17.26679
		Parsa,2017	Bupivacaine 2 mg/kg	12.98036
		Parsa,2017	Lidocaine 3 mg/kg	13.84923
		Parsa,2017	Lidocaine 6 mg/kg	12.77334
		Oztruk, 2010	Bupivacaine 2 mg/kg	14.78854
		Oztruk, 2010	Lidocaine 3 mg/kg	14.17087
		Oztruk, 2010	Prilocaine 7 mg/kg	14.17087
	Bupivacaine	Suckow,2011		34.35886
		Parsa,2017		31.79083
		Oztruk, 2010		33.85031
	Lidocaine	Parsa,2017_1		29.48071
		Parsa,2017_2		21.69293
		Oztruk, 2010		48.82636
Quantity	Total	Suckow,2011	Bupivacaine	16.7318
		Parsa,2017	Bupivacaine 2 mg/kg	13.48108
		Parsa,2017	Lidocaine 3 mg/kg	13.71491
		Parsa,2017	Lidocaine 6 mg/kg	13.04132
		Oztruk, 2010	Bupivacaine 2 mg/kg	14.82353
		Oztruk, 2010	Lidocaine 3 mg/kg	14.10368
		Oztruk, 2010	Prilocaine 7 mg/kg	14.10368
	Bupivacaine	Suckow,2011		35.36314
		Parsa,2017		31.46659
		Oztruk, 2010		33.17027
	Lidocaine	Parsa,2017_1		32.30682

		Parsa,2017_2		29.9045
		Oztruk, 2010		37.78867
Score	Total	Parsa,2017	Bupivacaine 2 mg/kg	9.274815
		Parsa,2017	Lidocaine 3mg/kg	10.63843
		Parsa,2017	Lidocaine 6mg/kg	9.028258
		Oztruk, 2010	Bupivacaine 2 mg/kg	12.86904
		Oztruk, 2010	Lidocaine 3 mg/kg	11.76732
		Oztruk, 2010	Prilocaine 7 mg/kg	11.59525
		Yuzbasioglu, 2008	EMLA1g	9.285212
		Yuzbasioglu, 2008	Lidocaine 1mg/kg	9.869057
		Kesici, 2024	Bupivacaine 2 mg/kg	7.544869
		Kesici, 2024	Levobupivacaine 2.5 mg/kg	8.127755
	Bupivacaine	Parsa,2017		31.90408
		Oztruk, 2010		28.23195
		Kesici, 2024		39.86397
	Lidocaine	Parsa,2017_1		23.77208
		Parsa,2017_2		20.4389
		Oztruk, 2010		30.08741
		Yuzbasioglu, 2008		25.70161
Severity	Total	Parsa,2017	Bupivacaine	18.95364
		Parsa,2017	Lidocaine 3mg/kg	12.62031
		Parsa,2017	Lidocaine 6mg/kg	15.18024
		Oztruk, 2010	Bupivacaine 2 mg/kg	18.67163
		Oztruk, 2010	Lidocaine 3 mg/kg	18.15968
		Oztruk, 2010	Prilocaine 7 mg/kg	16.41451
	Bupivacaine	Parsa,2017		49.25301

		Oztruk, 2010		50.74699
	Lidocaine	Parsa,2017_1		19.12206
		Parsa,2017_2		25.79782
		Oztruk, 2010		55.08011
Fibrosis	Total	Choi,2017_1	Lidocaine 0.5%	20.87663
		Choi,2017_1	Lidocaine 1%	21.34269
		Choi,2017_1	Lidocaine 2%	19.04747
		Choi,2017_1	Lidocaine 4%	17.59395
		Choi,2017_2	Lidocaine 4%	21.13926
Inflammation	Total	Choi,2017_1	Lidocaine 0.5%	20.57069
		Choi,2017_1	Lidocaine 1%	20.43733
		Choi,2017_1	Lidocaine 2%	19.31086
		Choi,2017_1	Lidocaine 4%	20.24057
		Choi,2017_2	Lidocaine 4%	19.44056

Supplementary Table S2. Definition of macroscopic and microscopic adhesion score

First author, publication year	Macroscopic adhesion score	Microscopic adhesion score
Yuzbasioglu 2008	**Cumulative adhesion scale**<br>+1 One adhesion band from the omentum to the target organ<br>+1 One adhesion band from the omentum to the abdominal scar<br>+1 One adhesion band from the omentum to anotber place<br>+1 One adhesion band from the adnexaJepididymal fat bodies to the target organ<br>+1 One adhesion band from the adnexafepididymal fat bodies to tbe abdominal scar<br>+1 One adhesion band from the adnexaJepididymal fat bodies to another place<br>+1 Any adhesive band other than described above<br>+1 Target organ adherent to the abdominal wall<br>+1 Target organ adherent to the abdominal scar<br>+1 Target organ adherent to the bowel<br>+1 Target organ adherent to the liver or the spleen<br>+1 Any other organ adherent	Not presented.
Oztruk 2010	**Extent**<br>0, no adhesions; 1, adhesions in 1–25 (%) of the total abraded area; 2, adhesions in 26–75 (%) of the total abraded area; 3, adhesions in 76–100 (%) of the total abraded area.<br>**Severity**<br>0, no adhesions; 1, adhesions lacking neovascularization; 2, thin, small vessels within adhesions; 3, significant vessels within adhesions.<br>**Quality**<br>0, no adhesions; 1, adhesions that can be detached with mild traction; 2, adhesions that can be detached with moderate<br>traction; 3, adhesions that can be detached with severe traction	Not presented.

Suckow 2011	**Extent**<br>0, no adhesion; 1, adhesion up to 25% of the implant surface; 2, adhesion up to 50% of the implant surface; 3, adhesion up to 70% of the implant surface; 4, adhesion up to 100% of the implant surface.<br>**Tenacity**<br>0, no resistance to separation; 1, mild resistance; 2, moderate resistance; 3, marked resistance; 4, sharp dissection required for separation.	Inflamation and inflammatory cell
Parsa 2017	**Extent**<br>0, no adhesion; 1, adhesion on to 25% of traumatized area; 2, adhesion on to 50% of traumatized area; 3, adhesion on to 70% of traumatized area.<br>**Severity**<br>0, no resistance to separation; 0.5, some resistance(moderate force required); 1, Sharp dissection needed; 1, Sharp dissection needed;	**Inflammation**<br>0, no inflammation; 1, mild inflammation; 2, moderate inflammation; 3, severe inflammation.
Choi 2017	Not presented.	**Inflammation**<br>0, no inflammation; 1, a few lymphocytes and plasma cells; 2, mild inflammatory infiltrate composed of lymphocytes, plasma cells, and polymorphonuclear leukocytes; 3, grade 2 plus neutrophils; 4, high concentrations (collections) of lymphocytes, plasma cells, polymorphonuclear leukocytes, histiocytes, and ulceration.<br>**Fibrosis**<br>0, no fibrosis; 1, mild fibrotic reaction around wound; 2, easily detected thick bands; 3, well-developed, dense collagen

		bands; 4, a severe fibrotic response replacing large areas.
Kesici 2024	Score<br>0, No adhesion; 1, Single band; 2, Double bands; 3, Multiple bands; 4, Direct visceral adhesion, cohesion	

Supplementary Table S3. Assessment of methodological quality.

First author, publication year	Statement of random allocation	Husbandry conditions	Compliance with animal welfare regulations-	Peer reviewed	Potential conflict of interest	Score
Yuzbasioglu 2008	1	1	1	1	1	5
Oztruk 2010	1	0	1	1	1	4
Suckow 2011	0	1	1	1	1	4
Parsa 2017	1	1	1	1	1	5
Choi 2017	1	1	1	1	1	5
Kesici 2024	0	1	1	1	1	4

Supplementary Table S4. Risk of bias assessed using SYRCLE's tool

First author, publication year	Item 1	Item 2	Item 3	Item 4	Item 5	Item 6	Item 7	Item 8	Item 9	Item 10
Yuzbasioglu 2008	Unclear [a]	Yes	Unclear [b]	Yes	Unclear [c]	Unclear [d]	Unclear [e]	Yes	Yes	Yes
Oztruk 2010	Unclear [a]	Yes	Unclear [b]	Yes	Unclear [c]	Unclear [d]	Unclear [e]	Yes	Yes	Yes
Suckow 2011	Unclear [a]	Yes	Unclear [b]	Yes	Unclear [c]	Unclear [d]	Unclear [e]	Yes	Yes	Yes
Parsa 2017	Unclear [a]	Yes	Unclear [b]	Yes	Unclear [c]	Unclear [d]	Unclear [e]	Yes	Yes	Yes
Choi 2017	Yes	Yes	Yes	Yes	Yes	Yes	Yes	Yes	Yes	Yes
Kesici 2024	Unclear [a]	Yes	Unclear [b]	Yes	Unclear [c]	Unclear [d]	Unclear [e]	Yes	Yes	Yes

Item	Description of domain
Item 1	Describe the methods used, if any, to generate the allocation sequence in sufficient detail to allow an assessment whether it should produce comparable groups.
Item 2	Describe all the possible prognostic factors or animal characteristics, if any, that are compared in order to judge whether or not intervention and control groups were similar at the start of the experiment.
Item 3	Describe the method used to conceal the allocation sequence in sufficient detail to determine whether intervention allocations could have been foreseen before or during enrolment.
Item 4	Describe all measures used, if any, to house the animals randomly within the animal room.
Item 5	Describe all measures used, if any, to blind trial caregivers and researchers from knowing which intervention each animal received. Provide any information relating to whether the intended blinding was effective.
Item 6	Describe whether or not animals were selected at random for outcome assessment, and which methods to select the animals, if any, were used.
Item 7	Describe all measures used, if any, to blind outcome assessors from knowing which intervention each animal received. Provide any information relating to whether the intended blinding was effective.
Item 8	Describe the completeness of outcome data for each main outcome, including attrition and exclusions from the analysis. State whether attrition and exclusions were reported, the numbers in each intervention group (compared with total randomized animals), reasons for attrition or exclusions, and any re-inclusions in analyses for the review.
Item 9	State how selective outcome reporting was examined and what was found.
Item 10	State any important concerns about bias not covered by other domains in the tool.

[a]: allocation sequence generation was not described in detail.
[b]: allocation concealment was not described
[c]: blinding of investigator was not described
[d]: animal selection at random for outcome assessment was not described in the manuscript
[e]: blinding of outcome assessor was not described

Supplementary Table S5. The GRADE evidence quality for each outcome assessed from pre-clinical studies

Outcome		Study limitations	Imprecision	Inconsistency	Indirectness	Publication bias	GRADE
Macroscopic	Quality (or tenacity of adhesion)	Serious [a]	Serious [b]	Serious [c]	Not serious	NA	⊕○○○ Very low
	Quantity (or extent of adhesion)	Serious [a]	Serious [b]	Serious [c]	Not serious	NA	⊕○○○ Very low
	Score	Serious [a]	Serious [b]	Not serious	Not serious	NA	⊕⊕○○ Low
Microscopic	Severity	Serious [a]	Serious [b]	Not serious	Not serious	NA	⊕⊕○○ Low
	Inflammation	Serious [a]	Serious [b]	Serious [c]	Not serious	NA	⊕○○○ Very low
	Fibrosis	Serious [a]	Serious [b]	Serious [c]	Not serious	NA	⊕○○○ Very low

NA: not assessed.

[a] We downgraded study limitation as includes studies included studies exhibited a high risk of bias.

[b] We downgraded imprecision by one level for serious imprecision due to very wide confidence intervals, including both substantial harms and benefits.

[c] We downgraded the certainty of evidence by one level for inconsistency due to heterogeneity among studies, with an I^2 statistic over 50% or P_{chi}^2 less than 0.05.
